# Performance of Machine Learning in Diagnosing KRAS (Kirsten Rat Sarcoma) Mutations in Colorectal Cancer: Systematic Review and Meta-Analysis

**DOI:** 10.2196/73528

**Published:** 2025-07-18

**Authors:** Kaixin Chen, Yin Qu, Ye Han, Yan Li, Huiyan Gao, De Zheng

**Affiliations:** 1 Department of Anorectal Surgery, Shuguang Hospital, Shanghai University of Traditional Chinese Medicine; Anorectal Disease Institute of Shuguang Hospital, 528 Zhangheng Road, Shanghai, 201203, China, 86 13641763662; 2 Department of Traditional Chinese Medicine Anorectal Surgery, Gongli Hospital of Shanghai Pudong New Area, Shanghai, China; 3 Traditional Chinese Medicine Department, Shanghai Pudong New Area Beicai Community Health Service Center, Pudong New Area, Shanghai, China

**Keywords:** colorectal cancer, Kirsten rat sarcoma viral oncogene, deep learning, machine learning, radiomics, PRISMA

## Abstract

**Background:**

With the widespread application of machine learning (ML) in the diagnosis and treatment of colorectal cancer (CRC), some studies have investigated the use of ML techniques for the diagnosis of KRAS (Kirsten rat sarcoma) mutation. Nevertheless, there is scarce evidence from evidence-based medicine to substantiate its efficacy.

**Objective:**

Our study was carried out to systematically review the performance of ML models developed using different modeling approaches, in diagnosing KRAS mutations in CRC. We aim to offer evidence-based foundations for the development and enhancement of future intelligent diagnostic tools.

**Methods:**

PubMed, Cochrane Library, Embase, and Web of Science were systematically retrieved, with the search cutoff date set to December 22, 2024. The encompassed studies are publicly published research papers that use ML to diagnose KRAS gene mutations in CRC. The risk of bias in the encompassed models was evaluated via the PROBAST (Prediction Model Risk of Bias Assessment Tool). A meta-analysis of the model’s concordance index (c-index) was performed, and a bivariate mixed-effects model was used to summarize sensitivity and specificity based on diagnostic contingency tables.

**Results:**

A total of 43 studies involving 10,888 patients were included. The modeling variables were derived from clinical characteristics, computed tomography (CT), magnetic resonance imaging (MRI), positron emission tomography/computed tomography, and pathological histology. In the validation cohort, for the ML model developed based on CT radiomic features, the c-index, sensitivity, and specificity were 0.87 (95% CI 0.84‐0.90), 0.85 (95% CI 0.80‐0.89), and 0.83 (95% CI 0.73‐0.89), respectively. For the model developed using MRI radiomic features, the c-index, sensitivity, and specificity were 0.77 (95% CI 0.71‐0.83), 0.78 (95% CI 0.72‐0.83), and 0.73 (95% CI 0.63‐0.81), respectively. For the ML model developed based on positron emission tomography/computed tomography radiomic features, the c-index, sensitivity, and specificity were 0.84 (95% CI 0.77‐0.90), 0.73, and 0.83, respectively. Notably, the deep learning (DL) model based on pathological images demonstrated a c-index, sensitivity, and specificity of 0.96 (95% CI 0.94‐0.98), 0.83 (95% CI 0.72‐0.91), and 0.87 (95% CI 0.77‐0.92), respectively. The DL model MRI-based model showed a c-index of 0.93 (95% CI 0.90‐0.96), sensitivity of 0.85 (95% CI 0.75‐0.91), and specificity of 0.83 (95% CI 0.77‐0.88).

**Conclusions:**

ML is highly accurate in diagnosing KRAS mutations in CRC, and DL models based on MRI and pathological images exhibit particularly strong diagnosis accuracy. More broadly applicable DL-based diagnostic tools may be developed in the future. However, the clinical application of DL models remains relatively limited at present. Therefore, future research should focus on increasing sample sizes, improving model architectures, and developing more advanced DL models to facilitate the creation of highly efficient intelligent diagnostic tools for KRAS mutation diagnosis in CRC.

## Introduction

Colorectal cancer (CRC) is the third most prevalent malignancy around the globe, with around 2 million new cases and 935,000 deaths in 2020, making up 10.7% of global cancer incidence and 9.5% of cancer-related mortality [1]. Its incidence significantly varies across different regions, at 36.4 per 100,000 population in America, 28.9 per 100,000 in Europe, and 28.8 per 100,000 in China [1,2]. Although the incidence has declined among the old in high-income countries, an upward trend is observed in emerging economies and among individuals under the age of 50 years worldwide [3]. This pattern reflects the critical public health challenge posed by CRC, prompting nations to intensify preventive efforts, promote early screening, and optimize therapeutic strategies to mitigate its societal and individual health impacts.

Currently, colorectal surgeries mainly involve the employment of laparoscopic minimally invasive techniques and robotic assistance [4], while modulation of the gut microbiota has proven effective in preventing anastomotic leakage [5]. Moreover, classical chemotherapy regimens combining oxaliplatin with 5-fluorouracil have demonstrated improved survival outcomes [6]. Immune checkpoint inhibitors exhibit specific efficacy in patients with mismatch repair deficiency [7], and targeted therapies aimed at suppressing A20 enhance immune responses and overcome drug resistance [8]. Despite continuous therapeutic advancements, overall patient prognosis remains suboptimal, with genetic mutations and molecular subtypes identified as key prognostic determinants [9]. Specifically, KRAS (Kirsten rat sarcoma) mutations are among the most common driver mutations, present in nearly 40% of CRC patients. Such mutations cause tumor cell activation, increase drug resistance, and are closely associated with poorer survival rates and diminished therapeutic efficacy [10]. However, current diagnostic methods have limitations. Tissue biopsy remains the gold standard for KRAS mutation diagnosis, yet its invasiveness and sampling constraints hinder comprehensive assessment of tumor heterogeneity, particularly in reflecting mutational discrepancies between primary and metastatic lesions [11]. Additionally, noninvasive circulating tumor DNA assays face sensitivity challenges in early-stage disease owing to low tumor DNA concentrations and dilution effects. Although combining circulating tumor DNA with trans-renal tumor DNA from urine samples may enhance diagnosis, such approaches are not yet widely adopted [12]. At the same time, emerging technologies such as surface-enhanced Raman scattering polymerase chain reaction and droplet digital polymerase chain reaction offer high sensitivity for KRAS mutation diagnosis but face limitations in clinical application owing to high costs, operational complexity, and insufficient clinical validation [13]. Therefore, there is an urgent need to explore novel methodologies to help with the diagnosis of KRAS mutations in CRC.

As artificial intelligence (AI) technologies advance, machine learning (ML) has garnered considerable attention from clinicians owing to its capability to integrate high-dimensional data effectively [14]. In clinical practice, ML is mainly used for biomarker diagnosis and prognostic analysis support [15]. As a critical subset of ML, deep learning (DL) excels in image data processing, significantly enhancing the diagnosis accuracy of cancer-specific survival in CRC histopathological analyses compared to traditional methods [16]. Some studies have used ML techniques to diagnose KRAS mutation status in CRC, revealing that DL can not only assess microsatellite instability to predict chemotherapy responses [17] but also integrate imaging, genomic, and clinical data to accurately diagnose KRAS mutations, thereby facilitating personalized targeted therapies [18]. Nevertheless, ML models encounter various challenges related to modeling discrepancies during application. The complexity and variability of clinical data can result in model performance instability and an increased risk of overfitting [19]. Differences in image data quality, resolution, and equipment can affect feature extraction and diagnosis capabilities, leading to poor performance on external datasets [20]. Moreover, limited pathological image annotation and heterogeneity constrain model training efficacy, leading to inconsistent predictions across different pathological subtypes [21].

At present, systematic evidence on how modeling variations impact the diagnosis of KRAS mutations in CRC remains limited. Therefore, this study aims to review the effectiveness of ML under varying modeling parameters and present evidence-based insights to facilitate the future application of AI technologies in CRC diagnosis and treatment.

## Methods

### Study Registration

This study was undertaken as per the PRISMA (Preferred Reporting Items for Systematic Reviews and Meta-Analyses, 2020) guidelines and has been prospectively registered on the PROSPERO (International Prospective Register of Systematic Reviews) platform (CRD42025610919) [22].

### Eligibility Criteria

The inclusion criteria were as follows:

1. Studies involving patients diagnosed with CRC;
2. Case-control, cohort, and cross-sectional studies about study designs;
3. An ML model was well constructed to differentiate KRAS mutation status. The model development process involved feature selection (via traditional ML techniques), model training, performance evaluation, and, where applicable, model validation; and
4. Publications reported in English.

The exclusion criteria were as follows:

1. Studies that fail to differentiate CRC from malignancies of other systems;
2. Meta-analyses, reviews, guidelines, expert opinions, or publicly available conference abstracts not subjected to peer review;
3. To evaluate the model’s performance, the study incorporated at least one key metric, such as the concordance index (c-index), sensitivity, specificity, accuracy, precision, confusion matrix, *F*_1_-score, or calibration curve, during the ML model construction process. If these metrics were absent, the model was excluded from the analysis; and
4. Those with an inadequate sample size (fewer than 20 cases). This requirement was set because of the stringent case number demands during model development. Typically, each additional variable necessitates an increase of 10 times in the number of positive cases (KRAS mutations), that is, ensuring an event per variable (EPV) greater than 10. Model development is generally based on 2 or more variables. However, when the number of cases is fewer than 20, the EPV requirement is not met.

### Data Sources and Search Strategy

The retrieval of PubMed, Cochrane Library, Embase, and Web of Science databases was thoroughly performed, covering studies published up to December 22, 2024. The search strategy integrated both Medical Subject Headings and free-text terms, with no restrictions on geographical region or publication year. The search strategies are detailed in Multimedia Appendix 1.

### Study Selection and Data Extraction

The retrieved studies were uploaded to EndNote (version 21.4, Thomson ResearchSoft). After duplicates were removed, titles and abstracts were checked to identify potentially eligible studies. Full-text papers were subsequently reviewed to determine final inclusion based on predefined criteria.

Before our data extraction, a standardized electronic data extraction form was developed, encompassing the variables as follows: first author, year of publication, country of the authors, study design, patient source, tumor staging, number of KRAS-mutant cases, total number of cases, approach of validation set generation, strategies to prevent overfitting, number of KRAS-mutant cases and total cases in the training and validation sets, sorts of models used, and variables used for model construction.

Data extraction was independently undertaken by 2 researchers (KC and DZ), which was followed by cross-verification. In cases of dissent, a third researcher (YQ) was asked for a decision.

### Risk of Bias in Studies

The risk of bias in eligible studies was assessed via the PROBAST (Prediction Model Risk of Bias Assessment Tool) [23], which evaluates 4 key domains: participants, predictors, outcomes, as well as analysis, to determine the overall risk of bias and applicability of these studies. Every domain comprises multiple specific items, with response options including “yes or probably yes” (low risk), “no or probably no” (high risk), and “no information” (unclear risk). A domain is categorized as high risk if any item within it is classified as high risk, and as low risk if all items are rated as low risk.

Furthermore, as our study incorporated a substantial amount of radiomics research, the radiomics quality score (RQS) was used for quality assessment. The RQS consists of 16 criteria, with a maximum score of 36 points. These criteria include: image protocol quality, multi-segmentation, modality studies, image acquisition time, feature dimensionality reduction, model construction using both omics and nonimaging features (prognosis and molecular subtyping), diagnosis and discussion of biological relevance, threshold analysis, discriminative statistics, calibration statistics, prospective studies registered in trial databases, validation, comparison with the “gold standard,” potential clinical utility, cost-benefit analysis, and open science or data availability [24].

The risk of bias assessment and quality assessment were independently carried out by 2 researchers (KC and DZ), with cross-checking undertaken after completion. All disagreements were settled through consultation with a third researcher (YQ).

### Synthesis Methods

A meta-analysis was undertaken on the c-index, a key metric for evaluating the overall accuracy of ML models. For studies lacking data on 95% CIs or SEs (SDs), SDs were estimated based on the methodology proposed by Debray et al [25]. Heterogeneity across studies was evaluated via the *I*² statistic. A random-effects model was applied if *I*² exceeded 50%, indicating substantial heterogeneity; otherwise, a fixed-effects model was used for *I*² values below 50%.

Furthermore, a meta-analysis of sensitivity and specificity was carried out via a bivariate mixed-effects model. This analysis was based on diagnostic 2×2 contingency tables. However, since these data were not reported directly in most primary studies, the necessary information was derived by integrating reported sensitivity, specificity, precision, and case numbers. The meta-analysis was enabled by Stata (version 15.1; StataCorp LLC).

## Results

### Study Selection

**Figure 1.**
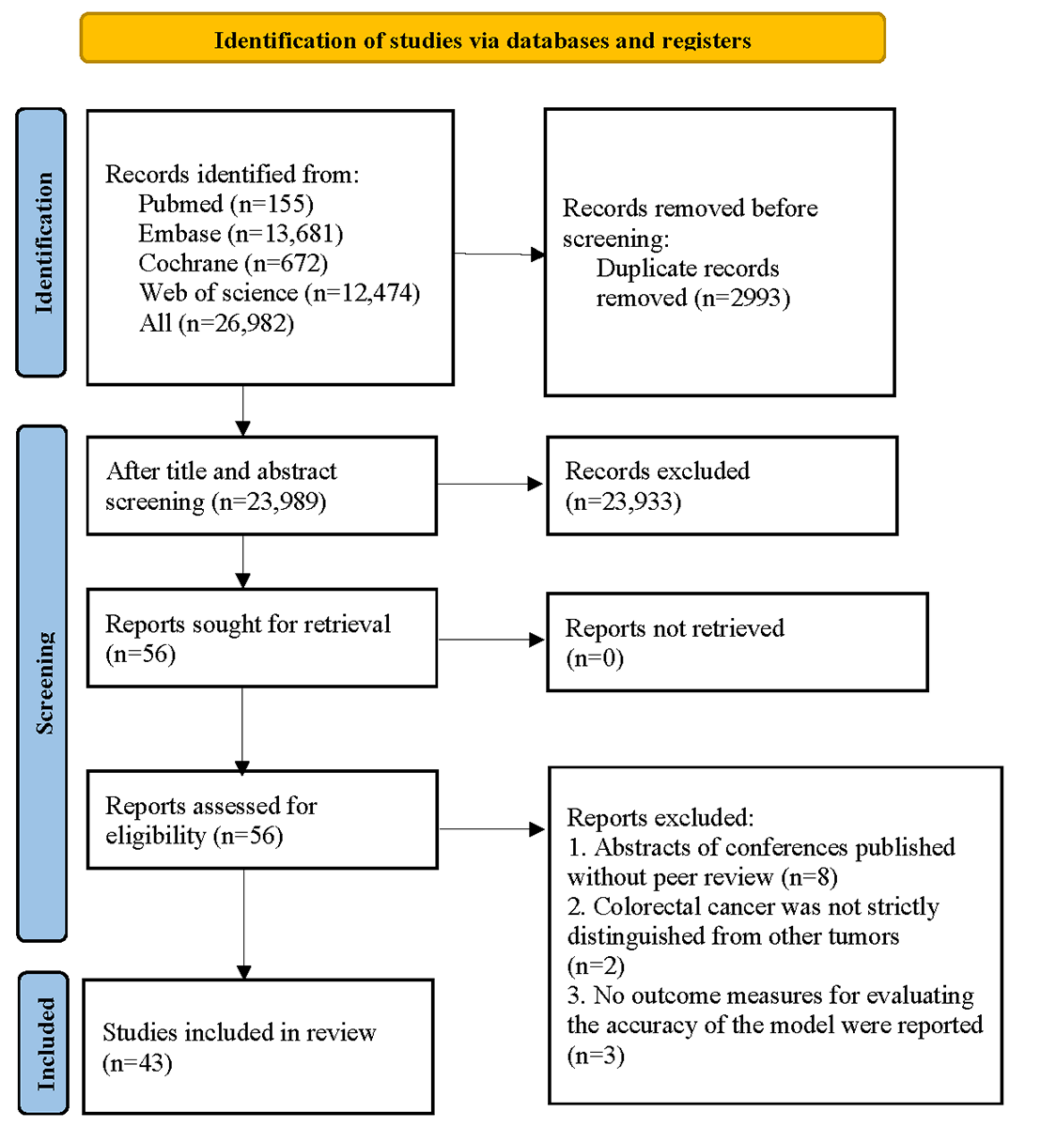
Literature screening process.

A total of 26,982 papers were retrieved after our literature search. After 2993 duplicates were removed, the titles and abstracts of the rest were meticulously screened, leading to the removal of 23,933 studies deemed irrelevant to the research topic. This initial screening process identified 56 potentially eligible articles. Following this, a thorough full-text evaluation was conducted. The full-text conference abstracts published without peer review did not rigorously distinguish between CRC and other cancers in the original research, nor did they report outcome measures assessing the accuracy of the evaluation models. Ultimately, 43 eligible studies were included in the final analysis [26-68] (Figure 1).

### Study Characteristics

Of the 43 eligible studies, all were case-control studies. Among them, 30 studies originated from China, 3 studies from South Korea, and the rest were conducted in countries such as the United States, Argentina, and France. Data from 27 studies were derived from single-center sources, 9 were multicenter studies, and 4 were based on registry databases, with some studies not explicitly reporting their data sources. Regarding the focus of the research, 12 studies concentrated on rectal cancer (RC), while the rest addressed CRC.

In terms of validation cohort generation, 33 studies provided relevant methodological details. The majority used random sampling, while some used cross-validation, internal validation, or external validation methods, with a minority applying the leave-one-out approach. The modeling variables were predominantly derived from computed tomography (CT) and magnetic resonance imaging (MRI) imaging data. Twelve studies developed models based on pathological slides or clinical characteristics. Additionally, 8 studies focused on multigene analyses involving KRAS, NRAS (neuroblastoma ras viral oncogene homolog), and BRAF (v-raf murine sarcoma viral oncogene homolog B), whereas the remaining studies concentrated on single-gene KRAS analyses. All characteristics of the included studies are detailed in Multimedia Appendix 2.

### Risk of Bias in Studies

A risk of bias assessment was performed for the 74 ML models encompassed in our review. Of these, 63 models used data from case-control studies that were not based on registry databases, reflecting a high risk of bias in study participant selection. Regarding predictor variables, since all models in this review were based on case-control studies, 4 models that relied on clinical characteristics may present a high risk of bias. However, most models were based on radiomics and pathological specimen data, which generally pose a lower risk of bias.

**Figure 2.**
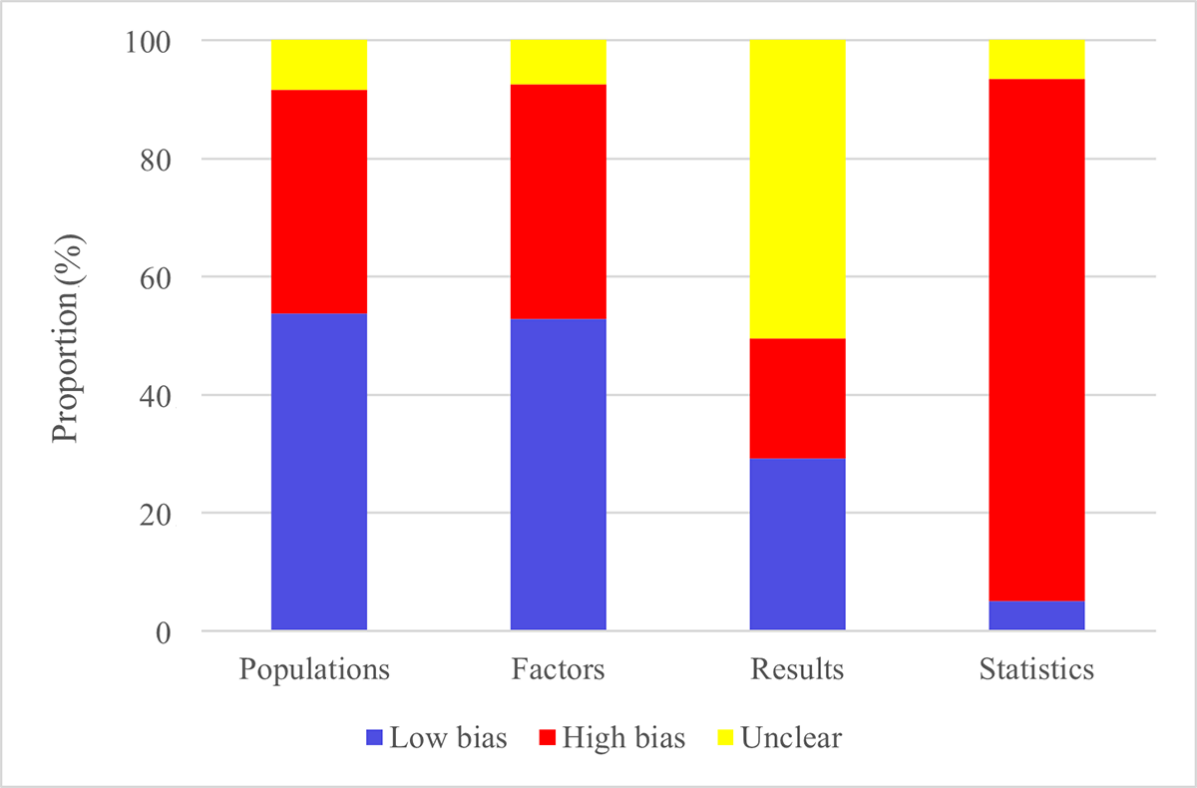
Risk assessment of the included models.

In our outcome assessment, as KRAS mutations were primarily diagnosed through pathological examinations serving as the gold standard, none of the encompassed models exhibited a risk of bias in this domain. Furthermore, for some ML models based on radiomics, the risk of bias related to case number selection remains unclear owing to the inability to derive the EPV ratio. Overall, data derived from radiomics and pathological specimens are relatively reliable, whereas data from nonregistry databases may affect the representativeness of study populations and introduce potential bias. Detailed risk of bias assessment results are presented in Figure 2.

Additionally, a quality assessment was conducted for the 33 radiomics studies included in the analysis. The results indicated that the following 6 items, “study of modality,” “image acquisition time,” “dimensionality reduction of features,” “diagnosis and discussion of biological relevance,” “prospective studies registered in trial databases,” and “comparison with the ‘gold standard’,” were not addressed in any of the studies, resulting in a score of 0 for these categories. All studies included a complete imaging protocol, thereby earning a score. Five studies did not perform multiple segmentations and received no score in this regard. Three studies explicitly stated the use of radiomics combined with clinical features for model construction and were accordingly awarded points. Eleven studies used threshold analysis, earning points for this criterion. All studies reported diagnostic statistics, resulting in full points for this item. Ten studies calibrated their statistical data, thereby receiving points. Most studies involved validation of their datasets, with multicenter studies scoring 3 points, single-center studies scoring 2 points, and 4 studies scoring 0 due to unclear validation methods. Eleven studies discussed the potential clinical utility of the models, earning points for this aspect. Only 1 study discussed cost-effectiveness analysis, and 1 point is thus earned. Four studies did not provide the scientific and data foundations for the models they developed, while the remaining studies offered open-source region-of-interest segmentation, gaining 1 point each. The final average score was 7.4 points (18.90%, SD 4.0; Multimedia Appendix 3).

### Meta-Analysis: KRAS Mutation

In the CRC training cohort, 36 models reported the c-index effect size for isolated KRAS mutations, with 34 models providing data directly extractable or indirectly calculable through 2*2 diagnostic tables. The c-index of models based on clinical characteristics was 0.69 (95% CI 0.68‐0.70), with sensitivity and specificity ranging from 0.66 to 0.79 and 0.57 to 0.58. For models based on CT radiomics, the c-index, sensitivity, and specificity were 0.86 (95% CI 0.82‐0.89), 0.92 (95% CI 0.84‐0.96), and 0.73 (95% CI 0.62‐0.82). Models using MRI presented a c-index of 0.73 (95% CI 0.65‐0.81), with a sensitivity and specificity of 0.75 (95% CI 0.68‐0.81) and 0.65 (95% CI 0.49‐0.79). Positron emission tomography/computed tomography (PET/CT)–enabled models showed a c-index of 0.66 (95% CI 0.52‐0.81), with sensitivity and specificity ranges of 0.62‐0.65 and 0.55‐0.80. Additionally, pathology-based models exhibited a c-index of 0.87 (95% CI 0.79‐0.94), sensitivity of 0.82 (95% CI 0.73‐0.88), and specificity of 0.81 (95% CI 0.70‐0.88; Tables1  2 and Figures S1-S5 in Multimedia Appendix 4).

**Table 1.** Meta-analysis of the concordance index (c-index) for ML^a^ diagnosis of MSI^b^ in CRC^c^.

Modeling variables and model types		Training set			Validation set		
		Models, n	c-index (95% CI)	*I* ^2^	Models, n	c-index (95% CI)	*I* ^2^
Clinical features							
	LR^d^	3	0.69 (0.68‐0.70)	0	3	0.64 (0.57‐0.71)	32.8
	DL^e^	—^f^	—	—	1	0.67 (0.57‐0.77)	—
	Overall	3	0.69 (0.68‐0.70)	0	4	0.65 (0.60‐0.70)	4.5
CT^g^							
	LR	9	0.81 (0.72‐0.91)	96.9	7	0.81 (0.70‐0.92)	94.5
	LASSO^h^	4	0.94 (0.89‐0.99)	91.7	3	0.87 (0.80‐0.93)	0
	RF^i^	1	0.98 (0.97‐1.00)	—	2	0.89 (0.69‐1.08)	84.1
	SVM^j^	1	0.86 (0.81‐0.91)	—	1	0.68 (0.53‐0.83)	—
	Boosting	2	0.89 (0.72‐1.05)	95.3	4	0.91 (0.85‐0.97)	86.3
	Ensemble	—	—	—	2	0.93 (0.82‐1.03)	78.6
	KNN^k^	1	0.67 (0.59‐0.75)	—	—	—	—
	Bayes	1	0.73 (0.65‐0.81)	—	—	—	—
	Overall	19	0.86 (0.82‐0.89)	96	19	0.87 (0.84‐0.90)	89.2
MRI^l^							
	LR	1	0.68 (0.60‐0.75)	—	3	0.74 (0.50‐0.98)	94.6
	LASSO	1	0.80 (0.69‐0.91)	—	2	0.68 (0.62‐0.75)	0
	LDA^m^				1	0.67 (0.58‐0.76)	
	ANN^n^	1	0.71 (0.63‐0.79)	—	1	0.68 (0.42‐0.94)	—
	SVM	1	0.72 (0.65‐0.79)	—	2	0.70 (0.62‐0.78)	0
	DT^o^	2	0.74 (0.47‐1.02)	96.7	2	0.61 (0.54‐0.68)	0
	DL	—	—	—	5	0.93 (0.90‐0.96)	70.5
	Overall	6	0.73 (0.65‐0.81)	85.3	16	0.77 (0.71‐0.83)	94.1
PET^p^/CT							
	LR	2	0.66 (0.52‐0.81)	72.7	—	—	—
	DL	—	—	—	1	0.84 (0.77‐0.90)	—
	Overall	2	0.66 (0.52‐0.81)	72.7	1	0.84 (0.77‐0.90)	—
Pathology							
	LR	1	0.77 (0.68‐0.86)	—	1	0.64 (0.40‐0.87)	—
	ANN	1	0.71 (0.65‐0.77)	—	—	—	—
	RF	2	0.93 (0.87‐1.00)	69.1	2	0.81 (0.72‐0.89)	0
	Boosting	2	0.92 (0.83‐1.01)	82.5	2	0.80 (0.68‐0.92)	0
	DL	—	—	—	4	0.96 (0.94‐0.98)	96.7
	Overall	6	0.87 (0.79‐0.94)	92.7	9	0.94 (0.91‐0.96)	93.4

a ML: machine learning.

b MSI: microsatellite instability.

c CRC: colorectal cancer.

d LR: logistic regression.

e DL: deep learning.

f Not applicable.

g CT: computed tomography.

h LASSO: least absolute shrinkage and selection operator.

i RF: random forest.

j SVM: support vector machine.

k KNN: k-nearest neighbors.

l MRI: magnetic resonance imaging.

m LDA: linear discriminant analysis.

n ANN: artificial neural network.

o DT: decision tree.

p PET: positron emission tomography.

**Table 2.** Meta-analysis of the sensitivity and specificity of ML^a^ in diagnosing MSI^b^ in CRC^c^.

Modeling variables and model types		Training set			Validation set		
		Models, n	Sen^d^ (95% CI)	Spe^e^ (95% CI)	Models, n	Sen (95% CI)	Spe (95% CI)
Clinical features							
	LR^f^	2	0.66‐0.79	0.57‐0.58	3	0.55‐0.79	0.47‐0.55
	DL^g^	—^h^	—	—	1	0.64	0.65
	Overall	2	0.66‐0.79	0.57‐0.58	4	0.67 (0.57‐0.76)	0.54 (0.47‐0.61)
CT^i^							
	LR	8	0.84 (0.76‐0.90)	0.77 (0.66‐0.85)	7	0.80 (0.74‐0.84)	0.72 (0.63‐0.79)
	LASSO^j^	4	0.97 (0.66‐1.00)	0.74 (0.36‐0.93)	3	0.71‐0.86	0.23‐1.00
	RF^k^	1	1.00	0.93	2	0.82‐0.85	0.75‐0.95
	SVM^l^	3	0.82‐0.86	0.71‐0.80	2	0.77‐0.78	0.63‐0.81
	Boosting	2	1.00‐1.01	0.18‐0.61	6	0.92 (0.84‐0.97)	0.90 (0.74‐0.97)
	KNN^m^	1	0.80	0.55	—	—	—
	Ensemble	—	—	—	2	0.93‐0.97	0.58‐0.93
	Overall	19	0.92 (0.84‐0.96)	0.73 (0.62‐0.82)	22	0.85 (0.80‐0.89)	0.83 (0.73‐0.89)
MRI^n^							
	LR	1	0.78	0.59	3	0.63‐0.88	0.45‐0.91
	LASSO	1	0.64	0.85	2	0.56‐0.58	0.64‐1.00
	DT^o^	2	0.83‐0.84	0.38‐0.8	2	0.71‐0.76	0.44‐0.54
	SVM	1	0.71	0.66	2	0.71‐0.72	0.63‐0.69
	LDA^p^	—	—	—	1	0.77	0.51
	DL	—	—	—	5	0.85 (0.75‐0.91)	0.83 (0.77‐0.88)
	Overall	5	0.75 (0.68‐0.81)	0.65 (0.49‐0.79)	15	0.78 (0.72‐0.83)	0.73 (0.63‐0.81)
PET^q^/CT							
	LR	2	0.62‐0.65	0.55‐0.80	—	—	—
	DL	—	—	—	1	0.73	0.83
	Overall	2	0.62‐0.65	0.55‐0.80	1	0.73	0.83
Pathology							
	LR	1	0.76	0.7	1	0.69	0.8
	ANN^r^	1	0.67	0.61	—	—	—
	RF	2	0.85‐0.89	0.80‐0.92	2	0.63‐0.83	0.80‐0.85
	Boosting	2	0.76‐0.92	0.84‐0.88	2	0.63‐0.75	0.90‐0.91
	DL	—	—	—	4	0.92 (0.83‐0.97)	0.88 (0.71‐0.96)
	Overall	6	0.82 (0.73‐0.88)	0.81 (0.70‐0.88)	9	0.83 (0.72‐0.91)	0.87 (0.77‐0.92)

a ML: machine learning.

b MSI: microsatellite instability.

c CRC: colorectal cancer.

d Sen: sensitivity.

e Spe: specificity.

f LR: logistic regression.

g DL: deep learning.

h Not applicable.

i CT: computed tomography.

j LASSO: least absolute shrinkage and selection operator.

k RF: random forest.

l SVM: support vector machine.

m KNN: k-nearest neighbors.

n MRI: magnetic resonance imaging.

o DT: decision tree.

p LDA: linear discriminant analysis.

q PET: positron emission tomography.

r ANN: artificial neural network.

In the validation cohort, the c-index was extracted from 49 models, while sensitivity and specificity were calculable from 51 models. The c-index, sensitivity, and specificity for clinical characteristic-based models were 0.65 (95% CI 0.60‐0.70), 0.67 (95% CI 0.57‐0.76), and 0.54 (95% CI 0.47‐0.61). For CT radiomics-based models, the corresponding values were 0.87 (95% CI 0.84‐0.90), 0.85 (95% CI 0.80‐0.89), and 0.83 (95% CI 0.73‐0.89). MRI-based models demonstrated a c-index of 0.77 (95% CI 0.71‐0.83), sensitivity of 0.78 (95% CI 0.72‐0.83), and specificity of 0.73 (95% CI 0.63‐0.81). PET/CT-based models had a c-index of 0.84 (95% CI 0.77‐0.90), with sensitivity and specificity of 0.73 and 0.83. Pathology-based models showed a c-index of 0.94 (95% CI 0.91‐0.96), sensitivity of 0.83 (95% CI 0.72‐0.91), and specificity of 0.87 (95% CI 0.77‐0.92). These findings indicate significant performance differences across data types, with pathology and CT radiomics models demonstrating superior diagnosis accuracy. It was found that models based solely on clinical features exhibited significantly lower diagnosis accuracy compared to those constructed using MRI, CT, and PET/CT radiomic features, as well as those built from pathological images. Furthermore, the model based on pathological images demonstrated the highest diagnosis accuracy (Tables1  2 and Figures S6-S10 in Multimedia Appendix 4).

For the RC training set, 7 models analyzed isolated KRAS mutations, yielding overall c-index, sensitivity, and specificity effect sizes of 0.77 (95% CI 0.63‐0.91), 0.77 (95% CI 0.67‐0.85), and 0.59 (95% CI 0.42‐0.74). In the validation cohort, 18 models provided a c-index of 0.76 (95% CI 0.71‐0.82), while sensitivity and specificity from 14 models were 0.78 (95% CI 0.70‐0.85) and 0.70 (95% CI 0.60‐0.79; Tables3  4 and Figures S11 and S12 in Multimedia Appendix 4).

Given the potential impact of model types on results, subgroup analyses were conducted. Some MRI and pathology-based models used DL algorithms. In the CRC MRI validation cohort, DL-based models demonstrated a c-index of 0.93 (95% CI 0.90‐0.96), sensitivity of 0.85 (95% CI 0.75‐0.91), and specificity of 0.83 (95% CI 0.77‐0.88). In pathology-based models, the c-index was 0.96 (95% CI 0.94‐0.98), with a sensitivity of 0.92 (95% CI 0.83‐0.97) and specificity of 0.88 (95% CI 0.71‐0.96).

For RC, the c-index effect size in the MRI validation cohort was calculated from 13 models, yielding 0.74 (95% CI 0.65‐0.82). Among these, 10 models provided sensitivity and specificity estimates of 0.75 (95% CI 0.67‐0.82) and 0.66 (95% CI 0.54‐0.75).

**Table 3.** Meta-analysis of the c-index^a^ for ML^b^ in diagnosing MSI^c^ in RC^d^.

Modeling variables	Training set			Validation set		
	Model, n	c-index (95% CI)	*I* ^2^	Model, n	c-index (95% CI)	*I* ^2^
Clinical features	—^e^	—	—	1	0.67 (0.57‐0.77)	—
MRI^f^	5	0.73 (0.64‐0.83)	88.2	13	0.74 (0.65‐0.82)	94.6
PET^g^/CT^h^	—	—	—	1	0.84 (0.77‐0.90)	—
CT	2	0.86 (0.61‐1.11)	95.6	1	0.81 (0.65‐0.98)	—
Pathology	—	—	—	2	0.88 (0.73‐1.04)	91.3
Overall	7	0.77 (0.63‐0.91)	97.3	18	0.76 (0.71‐0.82)	95.2

a c-index: concordance index.

b ML: machine learning.

c MSI: microsatellite instability.

d RC: rectal cancer.

e Not applicable.

f MRI: magnetic resonance imaging.

g PET: positron emission tomography.

h CT: computed tomography.

**Table 4.** Meta-analysis of the sensitivity and specificity of ML^a^ in diagnosing MSI^b^ in RC^c^.

Modeling variables	Training set			Validation set		
	Model, n	Sen^d^ (95% CI)	Spe^e^ (95% CI)	Model, n	Sen (95% CI)	Spe (95% CI)
Clinical features	—^f^	—	—	1	0.64	0.65
MRI^g^	5	0.75 (0.68‐0.81)	0.65 (0.49‐0.79)	10	0.75 (0.67‐0.82)	0.66 (0.54‐0.75)
PET^h^/CT^i^	—	—	—	1	0.73	0.83
CT	2	0.64‐0.1	0.22‐0.71	—	—	—
Pathology	—	—	—	2	0.91‐0.98	0.61‐0.94
Overall	7	0.77 (0.67‐0.85)	0.59 (0.42‐0.74)	14	0.78 (0.70‐0.85)	0.70 (0.60‐0.79)

a ML: machine learning.

b MSI: microsatellite instability.

c RC: rectal cancer.

d Sen: sensitivity.

e Spe: specificity.

f Not applicable.

g MRI: magnetic resonance imaging.

h PET: positron emission tomography.

i CT: computed tomography.

### KRAS in Conjunction With Other Gene Mutations

In the study of mixed genotypes, the training set included 5 models, with a c-index, sensitivity, and specificity of 0.87 (95% CI 0.77‐0.96), 0.89 (95% CI 0.77‐0.96), and 0.67 (95% CI 0.50‐0.80). In the validation set, the pooled c-index derived from 6 models was 0.90 (95% CI 0.84‐0.96), while the sensitivity and specificity, calculated from 4 models, were 0.83 (95% CI 0.73‐0.90) and 0.70 (95% CI 0.51‐0.84; Tables5  6 and Figures S13 and S14 in Multimedia Appendix 4).

**Table 5.** Meta-analysis of the c-index^a^ for ML^b^-based diagnosis of KRAS^c^ and other gene mutation status in CRC^d^ and RC^e^.

Modeling variables	Training set			Validation set		
	Model, n	c-index (95% CI)	*I* ^2^	Model, n	c-index (95% CI)	*I* ^2^
CT^f^	2	0.90 (0.79‐1.02)	72.6	1	0.79 (0.66‐0.92)	—^g^
Pathology	2	0.87 (0.68‐1.06)	98.3	1	0.82 (0.66‐0.99)	—
PET^h^/CT	1	0.76 (0.60‐0.92)	—	1	0.70 (0.48‐0.93)	—
MRI^i^	—	—	—	3	0.95 (0.91‐0.98)	39.5
Overall	5	0.87 (0.77‐0.96)	94.3	6	0.90 (0.84‐0.96)	69.4

a c-index: concordance index.

b ML: machine learning.

c KRAS: Kirsten rat sarcoma.

d CRC: colorectal cancer.

e RC: rectal cancer.

f CT: computed tomography.

g Not applicable.

h PET: positron emission tomography.

i MRI: magnetic resonance imaging.

**Table 6.** Meta-analysis of sensitivity and specificity for ML^a^-based diagnosis of KRAS^b^ and other gene mutation status in CRC^c^ and RC^d^.

Modeling variables	Training set			Validation set		
	Model, n	Sen^e^ (95% CI)	Spe^f^ (95% CI)	Model, n	Sen (95% CI)	Spe (95% CI)
CT^g^	2	0.90‐0.98	0.40‐0.64	1	0.89	0.45
Pathology	2	0.75‐0.91	0.66‐0.88	1	0.75	0.90
PET^h^/CT	1	0.81	0.69	1	0.79	0.55
MRI^i^	—^j^	—	—	1	0.85	0.80
Overall	5	0.89 (0.77‐0.96)	0.67 (0.50‐0.80)	4	0.83 (0.73‐0.90)	0.70 (0.51‐0.84)

a ML: machine learning.

b KRAS: Kirsten rat sarcoma.

c CRC: colorectal cancer.

d RC: rectal cancer.

e Sen: sensitivity.

f Spe: specificity.

g CT: computed tomography.

h PET: positron emission tomography.

i MRI: magnetic resonance imaging.

j Not applicable.

## Discussion

### Principal Findings

Our study proves that the KRAS mutation prediction model for CRC, built on ML, predominantly relies on radiomics and pathology, exhibiting high overall accuracy. Among these, the CT-based radiomics model shows a c-index of 0.87 (95% CI 0.84‐0.90), the MRI-based model has a c-index of 0.77 (95% CI 0.71‐0.83), while the pathology-based model achieves the highest c-index of 0.94 (95% CI 0.91‐0.96). Notably, the DL model significantly outperforms in terms of accuracy. In DL-based models, the MRI-based model has a c-index of 0.93 (95% CI 0.90‐0.96), with a sensitivity of 0.85 (95% CI 0.75‐0.91) and specificity of 0.83 (95% CI 0.77‐0.88). The pathology-based DL model performs exceptionally well, with a c-index of 0.96 (95% CI 0.94‐0.98), sensitivity of 0.92 (95% CI 0.83‐0.97), and specificity of 0.88 (95% CI 0.71‐0.96).

Previous reviews have highlighted certain advantages of DL-based KRAS mutation prediction for CRC. In 1 study, the highest c-index for the KRAS mutation in the validation set using ML was 0.58, while traditional pathological feature extraction methods face technical limitations. Additionally, the ambiguity surrounding gene mutation definitions restricts the training performance of AI models, leading to significant interpretative limitations of the results [69]. Conversely, Jia et al [70], based on radiomics, found the combined sensitivity, specificity, and c-index for the validation cohort (13 studies) to be 0.78 (95% CI 0.71‐0.84), 0.84 (95% CI 0.74‐0.90), and 0.86 (95% CI 0.83‐0.89). Subgroup analysis based on imaging modality and segmentation methods revealed a c-index of 0.84 (95% CI 0.80‐0.87), though the small sample size restricts the interpretation of the foregoing results. Furthermore, researchers have found that the co-occurrence of multiple gene mutations (eg, KRAS, NRAS, and BRAF) may further impact patient prognosis, yet relevant studies remain scarce and warrant further data support [71]. Overall, the studies included in the analysis featured small sample sizes, considerable variations in the sources of radiomics images and ML methods, and insufficient systematic subgroup analyses, which may influence the interpretation and generalizability of our findings. Given these limitations, our study further explores different modeling variables and ML model types to provide a more comprehensive description of the strengths of various modeling approaches, offering evidence to enhance the clinical application value of KRAS mutation prediction models in CRC.

It was noted that the included literature mainly focused on the application of radiomics and pathological slides. In radiomics, common diagnosis methods include CT colonography, MRI, and PET/CT. Clinically, CT colonography is a safe and noninvasive examination with a sensitivity of up to 93.8%, capable of accurately diagnosing adenomatous polyps (≥10 mm) and excelling in gastrointestinal anatomical assessment, preoperative staging, and the diagnosis of liver, lung, and abdominal cavity lesions, particularly in identifying calcified metastases [72,73]. In contrast, although MRI is the gold standard for randomized controlled trial staging and performs excellently in assessing liver metastasis, its high cost, longer examination time, and dependence on the operator’s technical proficiency limit its extensive application [73,74]. PET/CT, primarily used for preoperative assessment of complex cases or advanced patients, is not suitable as a routine screening tool owing to its high cost [74]. In general, CT colonography is important in CRC diagnosis and staging due to its efficiency, safety, and multifaceted capabilities. Our study found that CT radiomics is the predominant technology in the included studies, performing well in diagnosing KRAS mutations. However, it was also noted that current research in CT radiomics has not explored the application of DL. Future studies should focus on expanding sample sizes and investigating DL applications in CT imaging to enhance the precision and clinical value of KRAS mutation diagnosis.

Traditional ML relies on manually designed features, which often results in poor adaptability and the potential loss of important information. In contrast, DL uses convolutional neural networks for automatic extraction of image features, thereby retaining more information and enhancing both accuracy and robustness [75,76]. Furthermore, DL is well-suited for high-dimensional, nonlinear problems due to its reliance on large datasets and highly parameterized structures. When integrated with prior knowledge and data optimization, it can further improve accuracy and reliability [77]. In our study, DL is predominantly applied to the diagnosis of MRI and pathology-related specimens. Among these, MRI models based on DL demonstrated high diagnostic performance. Pathology models exhibited even higher c-index values. Additionally, in traditional ML, ensemble learning and boosting algorithms exhibited favorable performance on the validation set as well. However, the foregoing results are based on a limited sample size, which presents certain limitations. Therefore, future research should incorporate more image data to further optimize DL models and develop more advanced diagnosis tools.

In CRC diagnosis and treatment, mutations in key genetic loci such as NRAS and BRAF, in addition to KRAS mutations, are also of significant concern. Although NRAS mutations occur at a relatively low frequency in CRC, approximately 3%‐7%, they are highly valuable in comprehensive RAS gene testing before epidermal growth factor receptor–targeted therapy [78]. BRAF mutations not only significantly influence the effects of anti–epidermal growth factor receptor monoclonal antibody treatment but are also closely associated with tumor invasiveness and metastasis, making them crucial indicators for evaluating disease progression and treatment prognosis [79]. The study of mutations in these genes holds considerable potential for future exploration. In our research, some studies have combined KRAS mutations with other gene mutations such as NRAS and BRAF for overall gene mutation prediction analysis. Upon evaluation, these studies showed a certain degree of diagnostic accuracy. However, these studies did not strictly distinguish between specific mutated genotypes, which may reduce the credibility and clinical applicability of our results. Therefore, future research should further strengthen the application of ML in diagnosing mutations at various genetic loci, with a clear distinction of genotypes, to increase the scientific validity and clinical relevance of the models. This will provide more reliable evidence for early diagnosis, precise prevention, and customized CRC treatment strategies.

During the application of models in clinical practice, several challenges must be acknowledged. First, there is a challenge related to the source of the population. A significant number of studies to date are single-center studies, which introduce challenges in the generalizability and interpretation of the models constructed [80]. Second, the selection of modeling variables presents another challenge. In existing studies, the variables are typically categorized into clinical features and radiomics features. Clinical features may involve sensitive personal information during extraction, leading to concerns regarding the authenticity of clinical variables [81]. Concurrently, studies based on radiomics features face challenges in image segmentation, extraction, and filtering [82]. The segmentation process is often influenced by the researchers’ prior knowledge and experience, which may result in substantial variations in the segmented areas [83,84]. Additionally, radiomics-based research is reliant on image parameters, with differences in parameters between images that could affect image quality and, consequently, the generalizability of the constructed models [85,86]. Furthermore, during texture feature selection, there is a significant risk of information loss from the images [87], which poses challenges to the accuracy of the resulting models. It was also noted that the generation of validation sets in model construction often relies on internal validation from a single center, which significantly limits the generalizability of the models [88]. Finally, the sample size should be considered in model construction, which also reflects a significant challenge. Many studies had limited cases, which led to validation sets being generated primarily through cross-validation, or the absence of an independent validation set, thus constraining model interpretation. Therefore, the inclusion of more cases is suggested to address these challenges.

In the studies included in our review, the overall risk of bias, as assessed by PROBAST, appears to be relatively high. Moreover, the use of the RQS tool for evaluating radiomics studies still presents challenges. This is because both PROBAST and RQS are stringent assessment tools. While PROBAST is suitable for multivariable models used in diagnosis and prediction, it is often applied to retrospective case-control studies, with few prospective studies included [88]. As such, PROBAST’s evaluation results often indicate a high risk of bias, which is a significant challenge for diagnostic models. In our study, most of the included research pertains to diagnostic models, where bias may arise during case selection. Furthermore, the statistical analysis section mandates an EPV greater than 20, an independent validation set, and a validation set sample size greater than 100 for low bias. Many studies struggle to meet these stringent criteria, leading to a higher risk of bias in the statistical analysis [89]. Additionally, the statistical analysis should report whether the weights presented in the model align with those reported in practice [89]. This is particularly challenging for models with lower interpretability, such as neural networks, support vector machines, and XGBoost (Extreme Gradient Boosting), which often do not provide weights in the original validation set, making it difficult to assess consistency. These issues reflect that, while PROBAST is widely adopted to evaluate the risk of bias in ML, its criteria may be overly stringent for diagnostic models. Future research should consider updating the PROBAST tool accordingly [90].

Regarding the RQS assessment tool, it requires repeated experiments on images from different scanners, accounting for variations not only between different models of scanners but also between devices of the same model but from different manufacturers. Furthermore, repeated measurements at different time points are required. This is a stringent criterion, and it is difficult to accurately and effectively present such variations during the analysis, making it impossible to score these entries. Additionally, the tool requires prospective registration, with multiple iterations of downscaling, where successful registration adds 7 points, and downscaling adds 3 points, both contributing substantial weight. However, in practice, effective prospective registration is challenging to implement. For validation, it is difficult to conduct robust validation, and some studies may overlook this aspect. For these studies, failure to perform any form of validation results in a deduction of 7 points. From these perspectives, it is evident that RQS is a stringent scoring tool. Consequently, models evaluated using this tool face challenges in terms of quality. Moreover, many studies evaluating radiomics models with the RQS tool report relatively low scores [91,92].

### Advantages and Limitations of Our Study

The accuracy of KRAS mutation diagnosis via ML was comprehensively summarized across various modeling approaches. Our study also reveals that, currently, DL techniques have only been applied in the literature related to MRI and histopathology, while research in CT radiomics remains insufficient. This finding suggests that future research should further investigate the application of DL in CT radiomics to enhance the accuracy and clinical applicability of genetic testing, which holds significant research value and practical implications.

However, several limitations exist in this study. First, although subgroup analyses were conducted based on different modeling approaches, the limited number of eligible studies and a paucity of clear reporting of modeling details regarding training and validation sets in some studies led to an imbalance in the statistical representation of these sets. Second, the validation sets in the included studies were primarily generated through random sampling, which may impose certain limitations in interpreting model generalizability and performance. Furthermore, our design incorporated a comprehensive set of diagnosis variables for model construction. Therefore, during the registration process, we used the widely accepted PROBAST tool to assess the bias risk of the diagnosis model. However, throughout the research process, a significant number of radiomics models developed based on imaging data were identified. To address this, the RQS was used to assess the quality of the relevant literature, which introduces some discrepancies from the initial registration content. Furthermore, the encompassed studies in our analysis had small sample sizes, which possibly affected the representativeness of the results. Therefore, future research should incorporate larger datasets to ameliorate the stability and accuracy of the models.

### Conclusions

ML demonstrates ideal accuracy in diagnosing KRAS mutations in CRC, particularly DL models based on MRI and histopathological images. However, these conclusions are drawn from limited primary data. Future research should involve larger sample sizes and further optimize the development of more intelligent DL models to provide more precise and efficient tools for the intelligent diagnosis of CRC.

## Supplementary material

10.2196/73528 Multimedia Appendix 1 Literature search strategy.

10.2196/73528 Multimedia Appendix 2 Basic characteristics of included studies.

10.2196/73528 Multimedia Appendix 3 Radiomics quality score of the included models.

10.2196/73528 Multimedia Appendix 4 Additional figures file.

10.2196/73528 Checklist 1 PRISMA checklist.

## Abbreviations

AI: artificial intelligence
BRAF: v-raf murine sarcoma viral oncogene homolog B
c-index: concordance index
CRC: colorectal cancer
CT: computed tomography
DL: deep learning
EPV: event per variable
KRAS: Kirsten rat sarcoma
ML: machine learning
MRI: magnetic resonance imaging
NRAS: neuroblastoma ras viral oncogene homolog
PET/CT: positron emission tomography/computed tomography
PRISMA: Preferred Reporting Items for Systematic Reviews and Meta-Analyses
PROBAST: Prediction Model Risk of Bias Assessment Tool
PROSPERO: International Prospective Register of Systematic Reviews
RC: rectal cancer
RQS: radiomics quality score
XGBoost: Extreme Gradient Boosting
