# Supplementary material for: Performance of Machine Learning in Diagnosing KRAS (Kirsten Rat Sarcoma) Mutations in Colorectal Cancer: Systematic Review and Meta-Analysis
Source: J Med Internet Res. 2025 Jul 18;27:e73528. doi: 10.2196/73528 (PMC12294651; doi:10.2196/73528)
Supplement: Multimedia Appendix 1 [file jmir-v27-e73528-s001.docx]

**1. Pubmed**

| Search number | Query | Results |
| --- | --- | --- |
| #1 | Colorectal Neoplasms[MeSH Terms] | 155 |
| #2 | ((((((((((((((((((((((((((((((((((((Colorectal Neoplasms[Title/Abstract]) OR (Colorectal Neoplasm[Title/Abstract])) OR (Colorectal Tumors[Title/Abstract])) OR (Colorectal Tumor[Title/Abstract])) OR (Colorectal Cancer[Title/Abstract])) OR (Colorectal Cancers[Title/Abstract])) OR (Colorectal Carcinoma[Title/Abstract])) OR (Colorectal Carcinomas[Title/Abstract])) OR (Colonic Neoplasms[Title/Abstract])) OR (Colonic Neoplasm[Title/Abstract])) OR (Colon Neoplasms[Title/Abstract])) OR (Colon Neoplasm[Title/Abstract])) OR (Colon Cancers[Title/Abstract])) OR (Colonic Cancer[Title/Abstract])) OR (Colonic Cancers[Title/Abstract])) OR (Colon Cancer[Title/Abstract])) OR (Colon Adenocarcinoma[Title/Abstract])) OR (Colon Adenocarcinomas[Title/Abstract])) OR (Sigmoid Neoplasm[Title/Abstract])) OR (Sigmoid Cancer[Title/Abstract])) OR (Sigmoidal Cancer[Title/Abstract])) OR (Rectal Neoplasm[Title/Abstract])) OR (Rectum Neoplasms[Title/Abstract])) OR (Rectum Neoplasm[Title/Abstract])) OR (Rectal Tumors[Title/Abstract])) OR (Rectal Tumor[Title/Abstract])) OR (Rectum Cancers[Title/Abstract])) OR (Rectal Cancer[Title/Abstract])) OR (Rectal Cancers[Title/Abstract])) OR (Rectum Cancer[Title/Abstract])) OR (rectum tumour[Title/Abstract])) OR (retrorectal tumor[Title/Abstract])) OR (retrorectal tumour[Title/Abstract])) OR (rectal malignancy[Title/Abstract])) OR (rectal malignancies[Title/Abstract])) OR (rectum malignancy[Title/Abstract])) OR (rectum neoplasia[Title/Abstract]) | 11756 |
| #3 | (Colorectal Neoplasms[MeSH Terms] AND (booksdocs[Filter] OR clinicaltrial[Filter])) OR (((((((((((((((((((((((((((((((((((((Colorectal Neoplasms[Title/Abstract]) OR (Colorectal Neoplasm[Title/Abstract])) OR (Colorectal Tumors[Title/Abstract])) OR (Colorectal Tumor[Title/Abstract])) OR (Colorectal Cancer[Title/Abstract])) OR (Colorectal Cancers[Title/Abstract])) OR (Colorectal Carcinoma[Title/Abstract])) OR (Colorectal Carcinomas[Title/Abstract])) OR (Colonic Neoplasms[Title/Abstract])) OR (Colonic Neoplasm[Title/Abstract])) OR (Colon Neoplasms[Title/Abstract])) OR (Colon Neoplasm[Title/Abstract])) OR (Colon Cancers[Title/Abstract])) OR (Colonic Cancer[Title/Abstract])) OR (Colonic Cancers[Title/Abstract])) OR (Colon Cancer[Title/Abstract])) OR (Colon Adenocarcinoma[Title/Abstract])) OR (Colon Adenocarcinomas[Title/Abstract])) OR (Sigmoid Neoplasm[Title/Abstract])) OR (Sigmoid Cancer[Title/Abstract])) OR (Sigmoidal Cancer[Title/Abstract])) OR (Rectal Neoplasm[Title/Abstract])) OR (Rectum Neoplasms[Title/Abstract])) OR (Rectum Neoplasm[Title/Abstract])) OR (Rectal Tumors[Title/Abstract])) OR (Rectal Tumor[Title/Abstract])) OR (Rectum Cancers[Title/Abstract])) OR (Rectal Cancer[Title/Abstract])) OR (Rectal Cancers[Title/Abstract])) OR (Rectum Cancer[Title/Abstract])) OR (rectum tumour[Title/Abstract])) OR (retrorectal tumor[Title/Abstract])) OR (retrorectal tumour[Title/Abstract])) OR (rectal malignancy[Title/Abstract])) OR (rectal malignancies[Title/Abstract])) OR (rectum malignancy[Title/Abstract])) OR (rectum neoplasia[Title/Abstract]) AND (booksdocs[Filter] OR clinicaltrial[Filter])) | 14380 |
| #4 | machine learning[MeSH Terms] | 719 |
| #5 | (((((((((((((((((((((((((((((((((machine learning[Title/Abstract]) OR (artificial intelligence[Title/Abstract])) OR (Transfer Learning[Title/Abstract])) OR (Deep learning[Title/Abstract])) OR (Ensemble Learning[Title/Abstract])) OR (prediction model[Title/Abstract])) OR (random forest[Title/Abstract])) OR (neural network[Title/Abstract])) OR (neural networks[Title/Abstract])) OR (CNN[Title/Abstract])) OR (K-Nearest Neighbor[Title/Abstract])) OR (Support vector machine[Title/Abstract])) OR (SVM[Title/Abstract])) OR (Gradient Boosting Machine[Title/Abstract])) OR (Nomogram[Title/Abstract])) OR (XGBoost[Title/Abstract])) OR (Adaboost[Title/Abstract])) OR (LightGBM[Title/Abstract])) OR (CatBoost[Title/Abstract])) OR (Gradient Boosting[Title/Abstract])) OR (Decision tree[Title/Abstract])) OR (Regression Trees[Title/Abstract])) OR (ResNet[Title/Abstract])) OR (AlexNet[Title/Abstract])) OR (VGGNet[Title/Abstract])) OR (GoogLeNet[Title/Abstract])) OR (Naive Bayesian[Title/Abstract])) OR (Multilayer perceptron[Title/Abstract])) OR (Bayesian network[Title/Abstract])) OR (Radiomics[Title/Abstract])) OR (Radiomic[Title/Abstract])) OR (radiomics-based[Title/Abstract])) OR (radiomic signature[Title/Abstract])) OR (Texture[Title/Abstract]) | 5672 |
| #6 | (machine learning[MeSH Terms] AND (booksdocs[Filter] OR clinicaltrial[Filter])) OR ((((((((((((((((((((((((((((((((((machine learning[Title/Abstract]) OR (artificial intelligence[Title/Abstract])) OR (Transfer Learning[Title/Abstract])) OR (Deep learning[Title/Abstract])) OR (Ensemble Learning[Title/Abstract])) OR (prediction model[Title/Abstract])) OR (random forest[Title/Abstract])) OR (neural network[Title/Abstract])) OR (neural networks[Title/Abstract])) OR (CNN[Title/Abstract])) OR (K-Nearest Neighbor[Title/Abstract])) OR (Support vector machine[Title/Abstract])) OR (SVM[Title/Abstract])) OR (Gradient Boosting Machine[Title/Abstract])) OR (Nomogram[Title/Abstract])) OR (XGBoost[Title/Abstract])) OR (Adaboost[Title/Abstract])) OR (LightGBM[Title/Abstract])) OR (CatBoost[Title/Abstract])) OR (Gradient Boosting[Title/Abstract])) OR (Decision tree[Title/Abstract])) OR (Regression Trees[Title/Abstract])) OR (ResNet[Title/Abstract])) OR (AlexNet[Title/Abstract])) OR (VGGNet[Title/Abstract])) OR (GoogLeNet[Title/Abstract])) OR (Naive Bayesian[Title/Abstract])) OR (Multilayer perceptron[Title/Abstract])) OR (Bayesian network[Title/Abstract])) OR (Radiomics[Title/Abstract])) OR (Radiomic[Title/Abstract])) OR (radiomics-based[Title/Abstract])) OR (radiomic signature[Title/Abstract])) OR (Texture[Title/Abstract]) AND (booksdocs[Filter] OR clinicaltrial[Filter])) | 5704 |
| #7 | #3 AND #6 | 155 |

**2. Cochrane**

| Search number | Query | Results |
| --- | --- | --- |
| #1 | MeSH descriptor: [Colorectal Neoplasms] explode all trees | 13061 |
| #2 | (Colorectal Neoplasms):ti,ab,kw OR (Colorectal Neoplasm):ti,ab,kw OR (Colorectal Tumors):ti,ab,kw OR (Colorectal Tumor):ti,ab,kw OR (Colorectal Cancer):ti,ab,kw | 21235 |
| #3 | (Colorectal Cancers):ti,ab,kw OR (Colorectal Carcinoma):ti,ab,kw OR (Colorectal Carcinomas):ti,ab,kw OR (Colonic Neoplasms):ti,ab,kw OR (Colonic Neoplasm):ti,ab,kw | 6931 |
| #4 | (Colon Neoplasms):ti,ab,kw OR (Colon Neoplasm):ti,ab,kw OR (Colon Cancers):ti,ab,kw OR (Colonic Cancer):ti,ab,kw OR (Colonic Cancers):ti,ab,kw | 6331 |
| #5 | (Colon Cancer):ti,ab,kw OR (Colon Adenocarcinoma):ti,ab,kw OR (Colon Adenocarcinomas):ti,ab,kw OR (Sigmoid Neoplasm):ti,ab,kw OR (Sigmoid Cancer):ti,ab,kw | 7548 |
| #6 | (Sigmoidal Cancer):ti,ab,kw OR (Rectal Neoplasm):ti,ab,kw OR (Rectum Neoplasms):ti,ab,kw OR (Rectum Neoplasm):ti,ab,kw OR (Rectal Tumors):ti,ab,kw | 3967 |
| #7 | (Rectal Tumor):ti,ab,kw OR (Rectum Cancers):ti,ab,kw OR (Rectal Cancer):ti,ab,kw OR (Rectal Cancers):ti,ab,kw OR (Rectum Cancer):ti,ab,kw | 8818 |
| #8 | (rectum tumour):ti,ab,kw OR (retrorectal tumor):ti,ab,kw OR (retrorectal tumour):ti,ab,kw OR (rectal malignancy):ti,ab,kw OR (rectal malignancies):ti,ab,kw | 2340 |
| #9 | (rectum malignancy):ti,ab,kw OR (rectum neoplasia):ti,ab,kw | 214 |
| #10 | #1 or #2 or #3 or #4 or #5 or #6 or #7 or #8 or #9 | 31956 |
| #11 | MeSH descriptor: [Machine Learning] explode all trees | 1082 |
| #12 | (machine learning):ti,ab,kw OR (artificial intelligence):ti,ab,kw OR (Transfer Learning):ti,ab,kw OR (Deep learning):ti,ab,kw OR (Ensemble Learning):ti,ab,kw | 8180 |
| #13 | (prediction model):ti,ab,kw OR (random forest):ti,ab,kw OR (neural network):ti,ab,kw OR (neural networks):ti,ab,kw OR (CNN):ti,ab,kw | 10462 |
| #14 | (K-Nearest Neighbor):ti,ab,kw OR (Support vector machine):ti,ab,kw OR (SVM):ti,ab,kw OR (Gradient Boosting Machine):ti,ab,kw OR (Nomogram):ti,ab,kw | 2861 |
| #15 | (XGBoost):ti,ab,kw OR (Adaboost):ti,ab,kw OR (LightGBM):ti,ab,kw OR (CatBoost):ti,ab,kw OR (Gradient Boosting):ti,ab,kw | 345 |
| #16 | (Decision tree):ti,ab,kw OR (Regression Trees):ti,ab,kw OR (ResNet):ti,ab,kw OR (AlexNet):ti,ab,kw OR (VGGNet):ti,ab,kw | 1213 |
| #17 | (GoogLeNet):ti,ab,kw OR (Naive Bayesian):ti,ab,kw OR (Multilayer perceptron):ti,ab,kw OR (Bayesian network):ti,ab,kw OR (Radiomics):ti,ab,kw | 1251 |
| #18 | (Radiomic):ti,ab,kw OR (radiomics-based):ti,ab,kw OR (radiomic signature):ti,ab,kw OR (Texture):ti,ab,kw | 2623 |
| #19 | #11 or #12 or #13 or #14 or #15 or #16 or #17 or #18 | 21583 |
| #20 | #10 and #19 | 672 |

**3. Embase**

| Search number | Query | Results |
| --- | --- | --- |
| #1 | 'colorectal tumor'/exp | 510353 |
| #2 | 'colorectal neoplasms':ab,ti OR 'colorectal neoplasm':ab,ti OR 'colorectal tumors':ab,ti OR 'colorectal tumor':ab,ti OR 'colorectal cancer':ab,ti OR 'colorectal cancers':ab,ti OR 'colorectal carcinoma':ab,ti OR 'colorectal carcinomas':ab,ti OR 'colonic neoplasms':ab,ti OR 'colonic neoplasm':ab,ti OR 'colon neoplasms':ab,ti OR 'colon neoplasm':ab,ti OR 'colon cancers':ab,ti OR 'colonic cancer':ab,ti OR 'colonic cancers':ab,ti OR 'colon cancer':ab,ti OR 'colon adenocarcinoma':ab,ti OR 'colon adenocarcinomas':ab,ti OR 'sigmoid neoplasm':ab,ti OR 'sigmoid cancer':ab,ti OR 'sigmoidal cancer':ab,ti OR 'rectal neoplasm':ab,ti OR 'rectum neoplasms':ab,ti OR 'rectum neoplasm':ab,ti OR 'rectal tumors':ab,ti OR 'rectal tumor':ab,ti OR 'rectum cancers':ab,ti OR 'rectal cancer':ab,ti OR 'rectal cancers':ab,ti OR 'rectum cancer':ab,ti OR 'rectum tumour':ab,ti OR 'retrorectal tumor':ab,ti OR 'retrorectal tumour':ab,ti OR 'rectal malignancy':ab,ti OR 'rectal malignancies':ab,ti OR 'rectum malignancy':ab,ti OR 'rectum neoplasia':ab,ti | 352280 |
| #3 | 'machine learning'/exp | 536542 |
| #4 | 'machine learning':ab,ti OR 'artificial intelligence':ab,ti OR 'transfer learning':ab,ti OR 'deep learning':ab,ti OR 'ensemble learning':ab,ti OR 'prediction model':ab,ti OR 'random forest':ab,ti OR 'neural network':ab,ti OR 'neural networks':ab,ti OR cnn:ab,ti OR 'k-nearest neighbor':ab,ti OR 'support vector machine':ab,ti OR svm:ab,ti OR 'gradient boosting machine':ab,ti OR nomogram:ab,ti OR xgboost:ab,ti OR adaboost:ab,ti OR lightgbm:ab,ti OR catboost:ab,ti OR 'gradient boosting':ab,ti OR 'decision tree':ab,ti OR 'regression trees':ab,ti OR resnet:ab,ti OR alexnet:ab,ti OR vggnet:ab,ti OR googlenet:ab,ti OR 'naive bayesian':ab,ti OR 'multilayer perceptron':ab,ti OR 'bayesian network':ab,ti OR radiomics:ab,ti OR radiomic:ab,ti OR 'radiomics based':ab,ti OR 'radiomic signature':ab,ti OR texture:ab,ti | 489192 |
| #5 | #1 OR #2 | 543558 |
| #6 | #3 OR #4 | 748576 |
| #7 | #5 AND #6 | 13681 |

**4. Web of Science**

| Search number | Query | Results |
| --- | --- | --- |
| #1 | Colorectal Neoplasms (Topic) OR Colorectal Neoplasm (Topic) OR Colorectal Tumors (Topic) OR Colorectal Tumor (Topic) OR Colorectal Cancer (Topic) OR Colorectal Cancers (Topic) OR Colorectal Carcinoma (Topic) OR Colorectal Carcinomas (Topic) OR Colonic Neoplasms (Topic) OR Colonic Neoplasm (Topic) OR Colon Neoplasms (Topic) OR Colon Neoplasm (Topic) OR Colon Cancers (Topic) OR Colonic Cancer (Topic) OR Colonic Cancers (Topic) OR Colon Cancer (Topic) OR Colon Adenocarcinoma (Topic) OR Colon Adenocarcinomas (Topic) OR Sigmoid Neoplasm (Topic) OR Sigmoid Cancer (Topic) OR Sigmoidal Cancer (Topic) OR Rectal Neoplasm (Topic) OR Rectum Neoplasms (Topic) OR Rectum Neoplasm (Topic) OR Rectal Tumors (Topic) OR Rectal Tumor (Topic) OR Rectum Cancers (Topic) OR Rectal Cancer (Topic) OR Rectal Cancers (Topic) OR Rectum Cancer (Topic) OR rectum tumour (Topic) OR retrorectal tumor (Topic) OR retrorectal tumour (Topic) OR rectal malignancy (Topic) OR rectal malignancies (Topic) OR rectum malignancy (Topic) OR rectum neoplasia (Topic) | 400998 |
| #2 | machine learning (Topic) OR artificial intelligence (Topic) OR Transfer Learning (Topic) OR Deep learning (Topic) OR Ensemble Learning (Topic) OR prediction model (Topic) OR random forest (Topic) OR neural network (Topic) OR neural networks (Topic) OR CNN (Topic) OR K-Nearest Neighbor (Topic) OR Support vector machine (Topic) OR SVM (Topic) OR Gradient Boosting Machine (Topic) OR Nomogram (Topic) OR XGBoost (Topic) OR Adaboost (Topic) OR LightGBM (Topic) OR CatBoost (Topic) OR Gradient Boosting (Topic) OR Decision tree (Topic) OR Regression Trees (Topic) OR ResNet (Topic) OR AlexNet (Topic) OR VGGNet (Topic) OR GoogLeNet (Topic) OR Naive Bayesian (Topic) OR Multilayer perceptron (Topic) OR Bayesian network (Topic) OR Radiomics (Topic) OR Radiomic (Topic) OR radiomics-based (Topic) OR radiomic signature (Topic) OR Texture (Topic) | 2667261 |
| #3 | #2 AND #1 | 12474 |
