# Supplementary material for: Performance of Machine Learning in Diagnosing KRAS (Kirsten Rat Sarcoma) Mutations in Colorectal Cancer: Systematic Review and Meta-Analysis
Source: J Med Internet Res. 2025 Jul 18;27:e73528. doi: 10.2196/73528 (PMC12294651; doi:10.2196/73528)
Supplement: Multimedia Appendix 2 [file jmir-v27-e73528-s002.docx]

**Table S1**. Basic characteristics of included·studies.

| No | First author | Year of publication | Country of authors | Study type | Patient source | Tumor type | Gene mutation type | Number of mutation cases | Total number of cases | Total number of cases in training set | Method for Generation of validation set | Number of cases in validation set | Model type | Modeling variables |
| --- | --- | --- | --- | --- | --- | --- | --- | --- | --- | --- | --- | --- | --- | --- |
| 1 | Yulan Ma | 2024 | China | Retrospective | Single center | Colorectal cancer | KRAS |  | 306 |  | 5-fold cross-validation |  | Deep learning | T2W-MRI radiomics |
| 2 | Manman Li | 2024 | China | Retrospective | Multicenter | pI-pIV colorectal cancer | KRAS | 161 | 408 | 168 | Random sampling | 240 | Logistic regression | CT radiomics, clinical features |
| 3 | Zigui Huang | 2024 | China | Retrospective | Single center | 0-IV colorectal cancer | KRAS | 24 | 94 |  | Random sampling |  | Random forest | Clinical features |
| 4 | Meiling Cai | 2024 | China | Retrospective | Single center | Colorectal cancer | KRAS | 151 | 320 | 288 | Random sampling | 32 | Deep learning | T2W-MRI radiomics |
| 5 | Hongyue Zhao | 2024 | China | Retrospective | Single center | I-IV colorectal cancer | Genotype mixing | 35 | 62 | 37 | Random sampling | 25 | Support vector machine | PET/CT radiomics |
| 6 | Xuejie Li | 2024 | China | Retrospective | Single center | I-IV colorectal cancer | Genotype mixing | 69 | 129 | 103 | Random sampling | 26 | Deep learning | Histopathology |
| 7 | Nina Wesdorp | 2023 | The Netherlands | Retrospective | Multicenter | Colorectal cancer | KRAS | 194 | 384 | 204 | External validation Random sampling | 180 | Random forest, gradient boosting, gradient boosting + LightGBM, and integrated machine learning | Contrast-enhanced radiomics |
| 8 | Yuntai Cao | 2023 | China | Retrospective | Single center | cT 1-4 cN 0-2 colorectal cancer | KRAS | 207 | 447 | 313 | Random sampling | 134 | Random forest | Contrast-enhanced radiomics, clinical features |
| 9 | Mohammed S. Alshuhri | 2023 | Saudi Arabia | Retrospective | Single center | cT2-4 cN1-3 colorectal cancer | KRAS | 28 | 60 | 32 | 5-fold cross-validation | 28 | Neural network | T2W-MRI radiomics |
| 10 | Lin Zhao | 2023 | China | Retrospective | Single center | Colorectal cancer | KRAS | 151 | 320 | 288 | Random sampling | 32 | Deep learning | T2W-MRI radiomics |
| 11 | Yirong Xiang | 2023 | China | Retrospective | Multicenter | cT2-4 pT0-4 pN0-2 rectal cancer | KRAS | 36 | 103 | 73 | Random sampling | 30 | Support vector machine | T2W-MRI radiomics, CT radiomics |
| 12 | Kuochen Wu | 2023 | China | Retrospective | Single center | T 1-4 N 0-2 M 0-1 rectal cancer | KRAS | 82 | 160 | 128 | Random sampling | 32 | Deep learning | PET/CT radiomics |
| 13 | María Agustina Ricci Lara | 2023 | Argentina | Retrospective | Single center | Colorectal cancer | KRAS | 37 | 85 | 55 | 5-fold cross-validation | 30 | Ensemble learning | CT radiomics |
| 14 | Jacobo Porto-Álvarez | 2023 | Span | Retrospective | Single center | Colorectal cancer | KRAS | 30 | 56 |  | Cross-validation |  | AdaBoost, neural networks, decision trees, support vector machines, and random forests | Contrast-enhanced radiomics |
| 15 | Robin Heckenauer | 2023 | France | Retrospective | Registration database | Colon cancer | KRAS | 26 | 45 |  | 5-fold cross-validation |  | Deep learning | Histopathology |
| 16 | Jianfeng Hu | 2022 | China | Retrospective | Single center | I-IV colorectal cancer | KRAS | 101 | 231 | 184 | Random sampling | 47 | Logistic regression, support vector machine, and random trees | CT radiomics |
| 17 | Yimin Guo | 2022 | China | Retrospective | Registration database | Colorectal cancer | KRAS | 175 | 396 | 278 | Random sampling | 59 | Deep learning | Histopathology |
| 18 | Ting Xue | 2022 | China | Retrospective | Single center | T 1-4 N 0-3 colorectal cancer | KRAS | 67 | 172 | 121 | Random sampling | 51 | Logistic regression | CT radiomics |
| 19 | Huanhuan Liu | 2022 | China | Retrospective | Single center | Rectal cancer | KRAS | 157 | 376 | 288 | Random sampling | 88 | Deep learning Logistic regression | T2-MRI radiomics, clinical features |
| 20 | Waleed M Ghareeb | 2022 | Egypt | Retrospective | Registration databaseTCGA | Rectal cancer | KRAS | 54 | 751 |  | External validation |  | Deep learning | Histopathology |
| 21 | Filippo Crimì | 2022 | Italy | Retrospective | Single center | III-IV colorectal cancer | Genotype mixing | 18 | 47 |  |  |  |  | Contrast-enhanced radiomics |
| 22 | Yulan Ma | 2021 | China | Retrospective |  | Colorectal cancer | KRAS | 137 | 306 | 306 | 10-fold cross-validation | 39 | Deep learning | T2W-MRI radiomics |
| 23 | Chang Bian | 2021 | China | Retrospective | Multicenter | Colon cancer | Genotype mixing |  | 339 |  | Random sampling | 25 | Deep learning | Histopathology |
| 24 | Kai Song | 2021 | China | Retrospective | Registration database | Colorectal cancer | KRAS |  | 495 | 337 | Random sampling | 36 | Deep learning | T2-MRI radiomics |
| 25 | Zhiyuan Zhang | 2021 | China | Retrospective | Single center | I-III rectal cancer | KRAS | 41 | 83 | 59 | Random sampling | 24 | LASSO | T2-MRI radiomics |
| 26 | Guangwen Zhang | 2021 | China | Retrospective | Single center | pT 1-4 pN 0-1 rectal cancer | Genotype mixing | 53 | 202 | 108 |  | 94 | Deep learning | T2-MRI radiomics |
| 27 | Bum Sup Jang | 2021 | Korea | Retrospective | Single center | cT 2-4 cN 0-3 rectal cancer | KRAS | 30 | 110 | 110 | 10-fold cross-validation |  | Deep learning | CT radiomics |
| 28 | Hector Eduardo Sanchez-Ibarra | 2020 | Mexico | Retrospective | Multicenter | Colorectal cancer | KRAS | 225 | 500 | 146 | 5-fold cross-validation |  | Neural network | Histopathology |
| 29 | Ji Eun Oh | 2020 | Korea | Retrospective | Single center | Rectal cancer | KRAS | 25 | 60 |  |  |  | Decision tree | T2 weighting-MRI radiomics |
| 30 | Hyun Jong Jang | 2020 | Korea | Retrospective | Multicenter | Colorectal cancer | KRAS | 249 | 771 | 142 | External validation |  | Deep learning | Histopathology |
| 31 | Xiaofang Guo | 2020 | China | Retrospective | Single center | Rectal cancer | KRAS | 44 | 127 |  |  |  | Linear discriminant function | T2-MRI radiomics |
| 32 | Yanfen Cui | 2020 | China | Retrospective | Multicenter | pT 1-4 PN 0-2 rectal cancer | KRAS | 182 | 390 | 213 | Random sampling External validation | 177 | Logistic regression, decision tree and support vector machine | T2 weighting-MRI radiomics |
| 33 | Yu Li | 2020 | China | Retrospective | Single center | II-IV colorectal cancer | KRAS | 84 | 207 | 167 | Random sampling | 40 | Support vector machine | CT radiomics |
| 34 | Kan He | 2020 | China | Retrospective | Single center | I-IV colorectal cancer | KRAS | 74 | 157 | 117 | Random sampling | 40 | Random forest | CT radiomics |
| 35 | Zhuojun Yu | 2020 | China | Retrospective | Single center | I-IV  colorectal cancer | Genotype mixing | 96 | 276 |  | Bootstrap |  | Logistic regression | Clinical features |
| 36 | Xiaomei Wu | 2020 | China | Retrospective | Single center | Colorectal cancer | KRAS | 197 | 398 | 279 | External validation | 119 | Multivariate logistic regression | CT radiomics |
| 37 | Jiawen Wang | 2020 | China | Retrospective | Single center | Rectal cancer | KRAS |  | 306 |  | 20-fold cross-validation |  | Deep learning | T2 weighting-MRI radiomics |
| 38 | Ruichuan Shi | 2020 | China | Retrospective | Multicenter | Colorectal cancer | Genotype mixing | 82 | 159 | 124 | Random sampling | 35 | Neural network | CT radiomics |
| 39 | Narumi Taguchi | 2019 | Japan | Retrospective | Single center | II-IV colorectal cancer | KRAS | 20 | 40 |  | 5-fold cross-validation |  | Logistic regression, support vector machine | PET/CT radiomics |
| 40 | Xiaochun Meng | 2019 | China | Retrospective | Single center | Rectal cancer | KRAS | 99 | 345 | 197 | External validation (prospective) | 148 | LASSO regression, random forest, support vector machine | MP-MRI radiomics |
| 41 | Lei Yang | 2018 | China | Retrospective | Single center | I-IV colorectal cancer | Genotype mixing | 72 | 117 | 61 | External validation (prospective) | 56 | Support vector machine | Contrast-enhanced radiomics |
| 42 | Shangwen Chen | 2018 | China | Retrospective | Single center | I-IV colorectal cancer | KRAS | 21 | 74 |  |  |  | Logistic regression | PET/CT radiomics |
| 43 | Yash Pershad | 2017 | America | Retrospective | Single center | Colon cancer | KRAS | 152 | 299 | 299 |  |  | Naive Bayes | Clinical features |
