# Supplementary material for: Performance of Machine Learning in Diagnosing KRAS (Kirsten Rat Sarcoma) Mutations in Colorectal Cancer: Systematic Review and Meta-Analysis
Source: J Med Internet Res. 2025 Jul 18;27:e73528. doi: 10.2196/73528 (PMC12294651; doi:10.2196/73528)
Supplement: Multimedia Appendix 3 [file jmir-v27-e73528-s003.docx]

| Year of publication | v1 | v2 | v3 | v4 | v5 | v6 | v7 | v8 | v9 | v10 | v11 | v12 | v13 | v14 | v15 | v16 | Total Score | Proportion |
| --- | --- | --- | --- | --- | --- | --- | --- | --- | --- | --- | --- | --- | --- | --- | --- | --- | --- | --- |
| 2024 | 1 | 1 | 0 | 0 | 0 | 0 | 0 | 0 | 2 | 0 | 0 | 3 | 0 | 0 | 0 | 1 | 8 | 22.22 |
| 2024 | 1 | 1 | 0 | 0 | 0 | 1 | 0 | 1 | 2 | 2 | 0 | 3 | 0 | 2 | 0 | 1 | 14 | 38.89 |
| 2024 | 1 | 1 | 0 | 0 | 0 | 0 | 0 | 0 | 2 | 0 | 0 | 2 | 0 | 0 | 0 | 1 | 7 | 19.44 |
| 2024 | 1 | 0 | 0 | 0 | 0 | 0 | 0 | 0 | 2 | 2 | 0 | 2 | 0 | 0 | 0 | 1 | 8 | 22.22 |
| 2023 | 1 | 1 | 0 | 0 | 0 | 0 | 0 | 0 | 1 | 2 | 0 | 3 | 0 | 0 | 0 | 1 | 9 | 25.00 |
| 2023 | 1 | 1 | 0 | 0 | 0 | 1 | 0 | 0 | 1 | 2 | 0 | 2 | 0 | 2 | 0 | 1 | 11 | 30.56 |
| 2023 | 1 | 1 | 0 | 0 | 0 | 0 | 0 | 0 | 1 | 0 | 0 | 2 | 0 | 0 | 0 | 1 | 6 | 16.67 |
| 2023 | 1 | 1 | 0 | 0 | 0 | 0 | 0 | 0 | 1 | 0 | 0 | 2 | 0 | 0 | 0 | 1 | 6 | 16.67 |
| 2023 | 1 | 1 | 0 | 0 | 0 | 0 | 0 | 1 | 1 | 0 | 0 | 3 | 0 | 2 | 0 | 0 | 9 | 25.00 |
| 2023 | 1 | 1 | 0 | 0 | 0 | 0 | 0 | 0 | 1 | 0 | 0 | 2 | 0 | 0 | 0 | 1 | 6 | 16.67 |
| 2023 | 1 | 1 | 0 | 0 | 0 | 0 | 0 | 0 | 1 | 2 | 0 | 2 | 0 | 0 | 0 | 1 | 8 | 22.22 |
| 2023 | 1 | 0 | 0 | 0 | 0 | 0 | 0 | 0 | 1 | 0 | 0 | 2 | 0 | 0 | 0 | 1 | 5 | 13.89 |
| 2022 | 1 | 1 | 0 | 0 | 0 | 0 | 0 | 0 | 2 | 2 | 0 | 2 | 0 | 2 | 0 | 1 | 11 | 30.56 |
| 2022 | 1 | 1 | 0 | 0 | 0 | 0 | 0 | 1 | 2 | 2 | 0 | 2 | 0 | 2 | 0 | 1 | 12 | 33.33 |
| 2022 | 1 | 1 | 0 | 0 | 0 | 1 | 0 | 0 | 2 | 2 | 0 | 2 | 0 | 2 | 0 | 1 | 12 | 33.33 |
| 2022 | 1 | 1 | 0 | 0 | 0 | 0 | 0 | 0 | 1 | 0 | 0 | -5 | 0 | 0 | 0 | 1 | -1 | -2.78 |
| 2021 | 1 | 1 | 0 | 0 | 0 | 0 | 0 | 0 | 2 | 0 | 0 | 2 | 0 | 0 | 0 | 1 | 7 | 19.44 |
| 2021 | 1 | 1 | 0 | 0 | 0 | 0 | 0 | 0 | 2 | 0 | 0 | 5 | 0 | 0 | 0 | 1 | 10 | 27.78 |
| 2021 | 1 | 1 | 0 | 0 | 0 | 0 | 0 | 1 | 1 | 0 | 0 | 2 | 0 | 2 | 0 | 0 | 8 | 22.22 |
| 2021 | 1 | 1 | 0 | 0 | 0 | 0 | 0 | 0 | 2 | 0 | 0 | 2 | 0 | 0 | 0 | 1 | 7 | 19.44 |
| 2021 | 1 | 1 | 0 | 0 | 0 | 0 | 0 | 1 | 2 | 0 | 0 | 2 | 0 | 0 | 0 | 1 | 8 | 22.22 |
| 2020 | 1 | 1 | 0 | 0 | 0 | 0 | 0 | 0 | 1 | 0 | 0 | -5 | 0 | 0 | 0 | 1 | -1 | -2.78 |
| 2020 | 1 | 1 | 0 | 0 | 0 | 0 | 0 | 1 | 1 | 0 | 0 | -5 | 0 | 0 | 0 | 0 | -1 | -2.78 |
| 2020 | 1 | 1 | 0 | 0 | 0 | 0 | 0 | 1 | 2 | 2 | 0 | 3 | 0 | 2 | 0 | 1 | 13 | 36.11 |
| 2020 | 1 | 1 | 0 | 0 | 0 | 0 | 0 | 0 | 2 | 0 | 0 | 2 | 0 | 2 | 0 | 1 | 9 | 25.00 |
| 2020 | 1 | 1 | 0 | 0 | 0 | 0 | 0 | 0 | 2 | 0 | 0 | 2 | 0 | 0 | 0 | 1 | 7 | 19.44 |
| 2020 | 1 | 1 | 0 | 0 | 0 | 0 | 0 | 1 | 2 | 2 | 0 | 2 | 0 | 2 | 1 | 1 | 13 | 36.11 |
| 2020 | 1 | 1 | 0 | 0 | 0 | 0 | 0 | 0 | 2 | 0 | 0 | 2 | 0 | 0 | 0 | 1 | 7 | 19.44 |
| 2020 | 1 | 1 | 0 | 0 | 0 | 0 | 0 | 1 | 1 | 0 | 0 | 3 | 0 | 2 | 0 | 1 | 10 | 27.78 |
| 2019 | 1 | 0 | 0 | 0 | 0 | 0 | 0 | 0 | 2 | 0 | 0 | 2 | 0 | 0 | 0 | 1 | 6 | 16.67 |
| 2019 | 1 | 0 | 0 | 0 | 0 | 0 | 0 | 1 | 1 | 0 | 0 | 2 | 0 | 0 | 0 | 1 | 6 | 16.67 |
| 2018 | 1 | 0 | 0 | 0 | 0 | 0 | 0 | 1 | 2 | 0 | 0 | 2 | 0 | 0 | 0 | 1 | 7 | 19.44 |
| 2018 | 1 | 1 | 0 | 0 | 0 | 0 | 0 | 0 | 1 | 0 | 0 | -5 | 0 | 0 | 0 | 0 | -2 | -5.56 |
