# Supplementary material for: Performance of Machine Learning in Diagnosing KRAS (Kirsten Rat Sarcoma) Mutations in Colorectal Cancer: Systematic Review and Meta-Analysis
Source: J Med Internet Res. 2025 Jul 18;27:e73528. doi: 10.2196/73528 (PMC12294651; doi:10.2196/73528)
Supplement: Multimedia Appendix 4 [file jmir-v27-e73528-s004.docx]

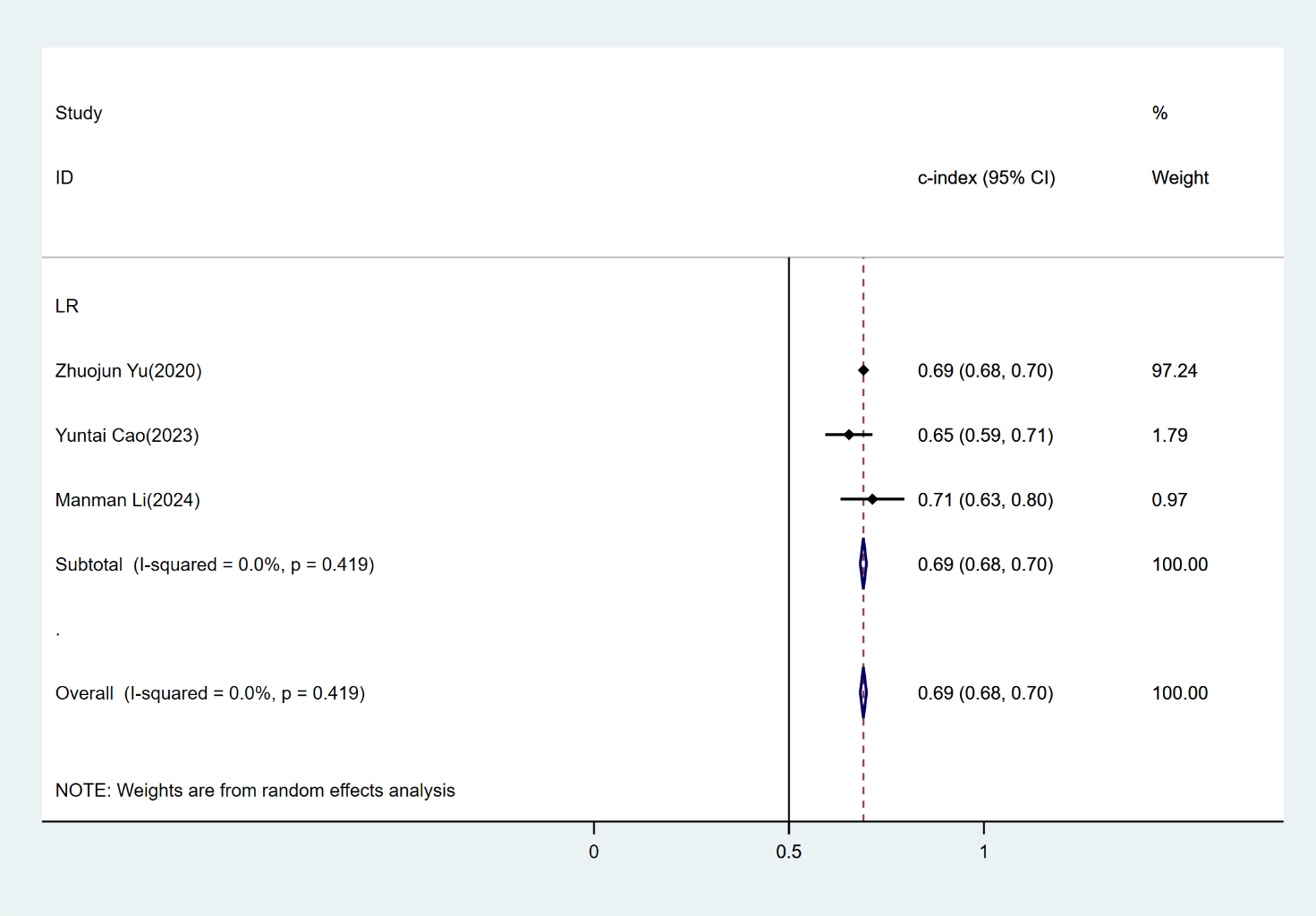


**Figure S1.** Forest plot of the c-index for detecting microsatellite instability in colorectal cancer based on clinical features in the training set.


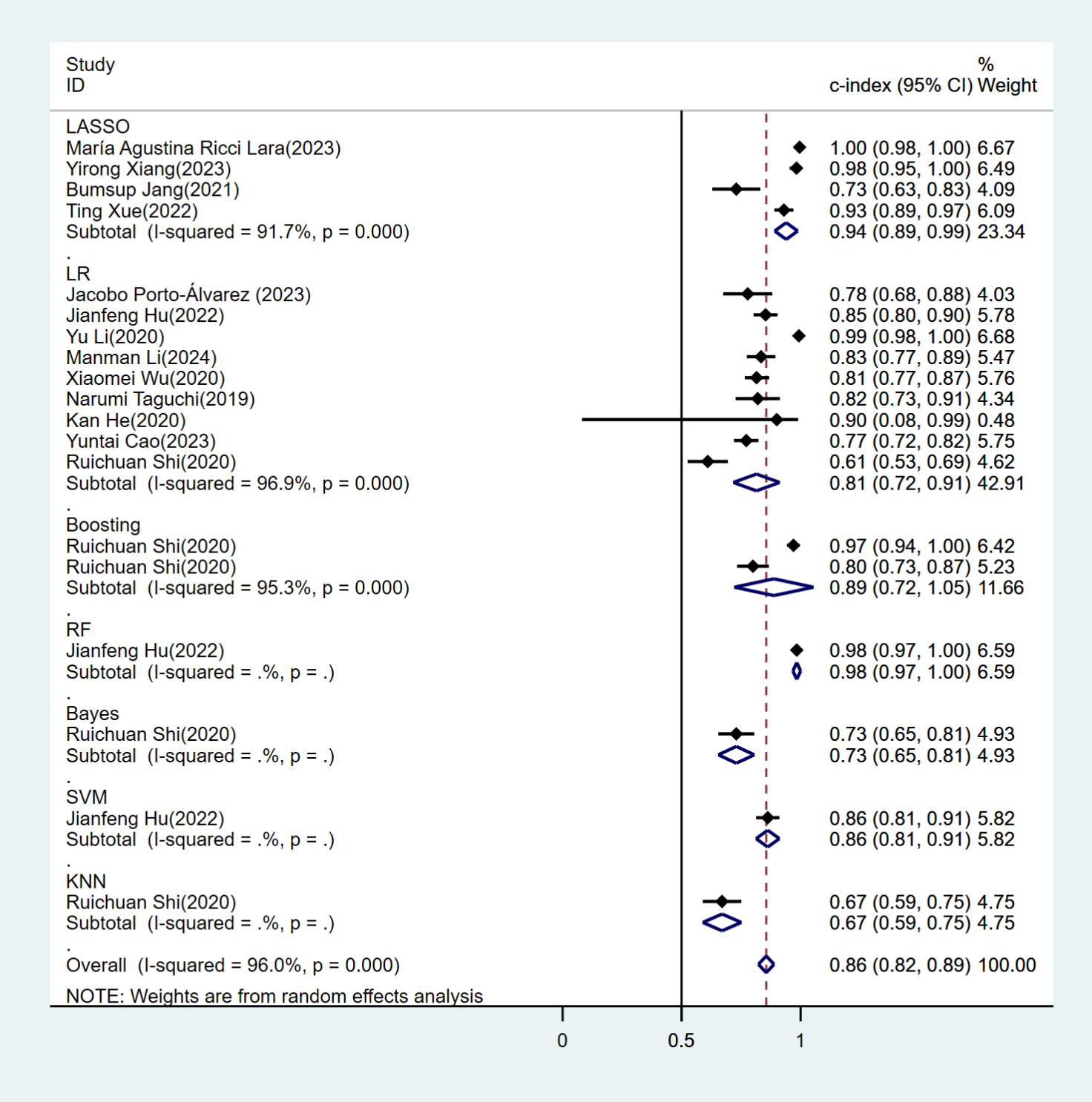


**Figure S2.** Forest plot of the c-index for detecting microsatellite instability in colorectal cancer based on CT radiomics in the training set.


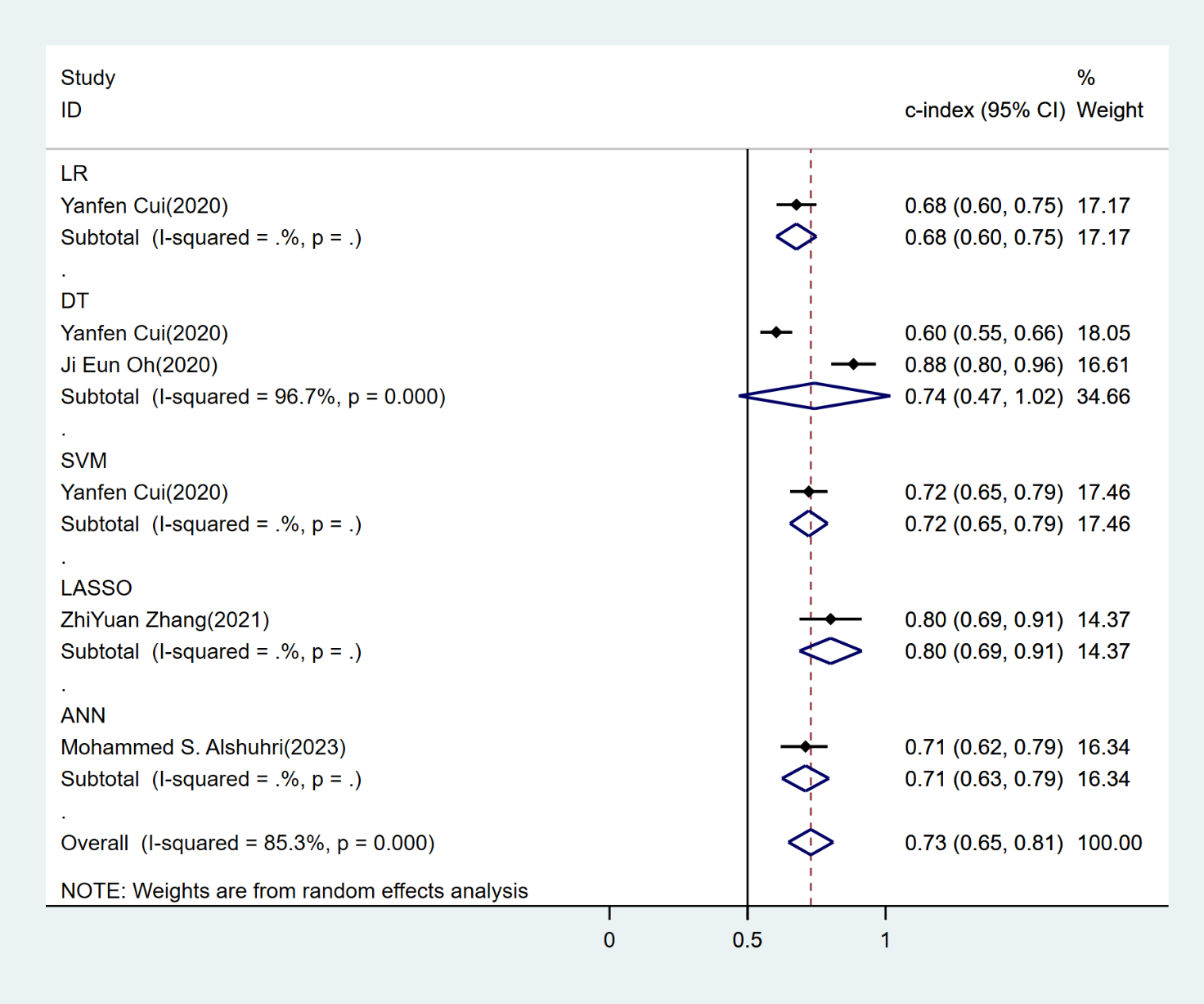


**Figure S3.** Forest plot of the c-index for detecting microsatellite instability in colorectal cancer based on MRI radiomics in the training set.
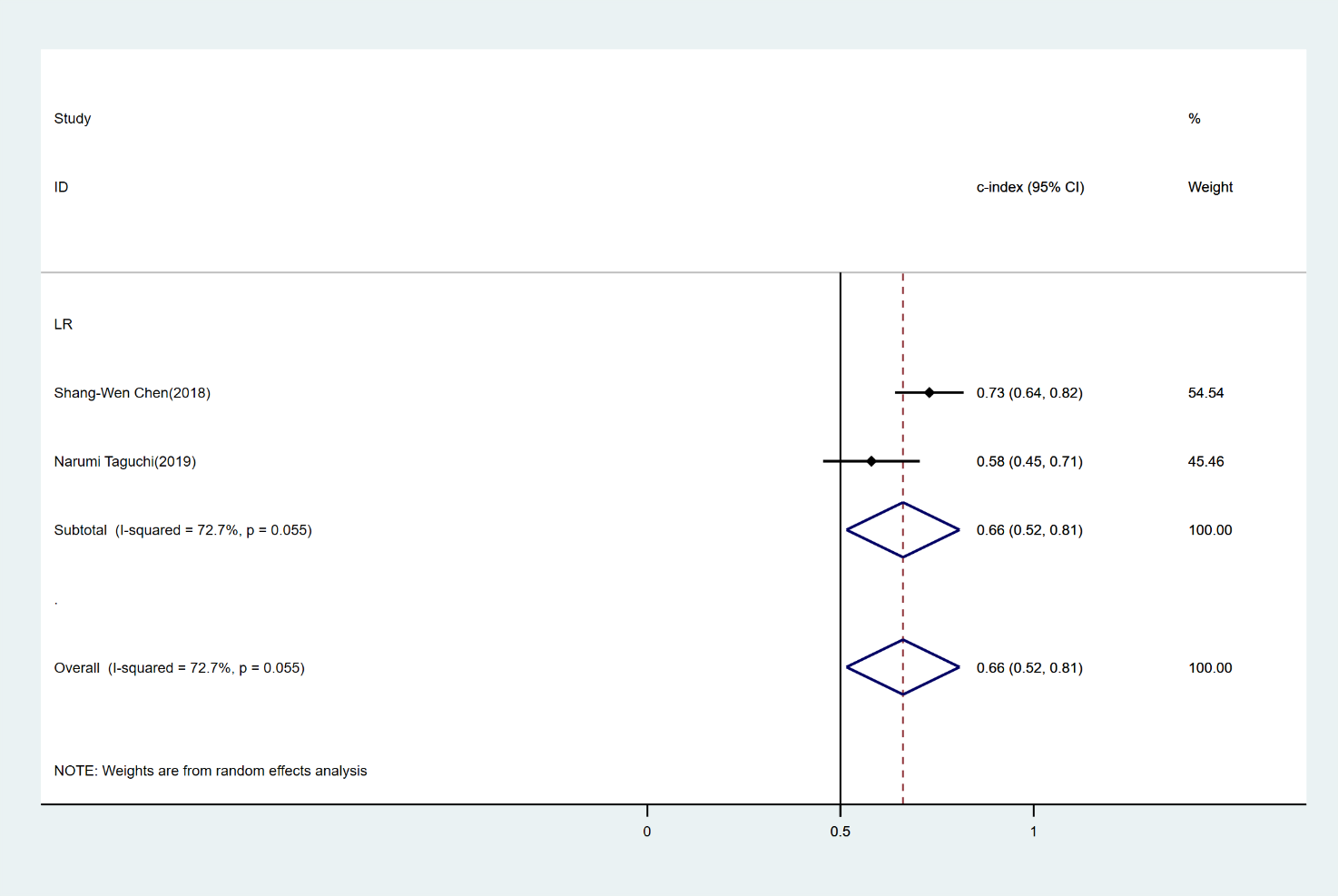


**Figure S4.** Forest plot of the c-index for detecting microsatellite instability in colorectal cancer based on PET/CT radiomics in the training set.


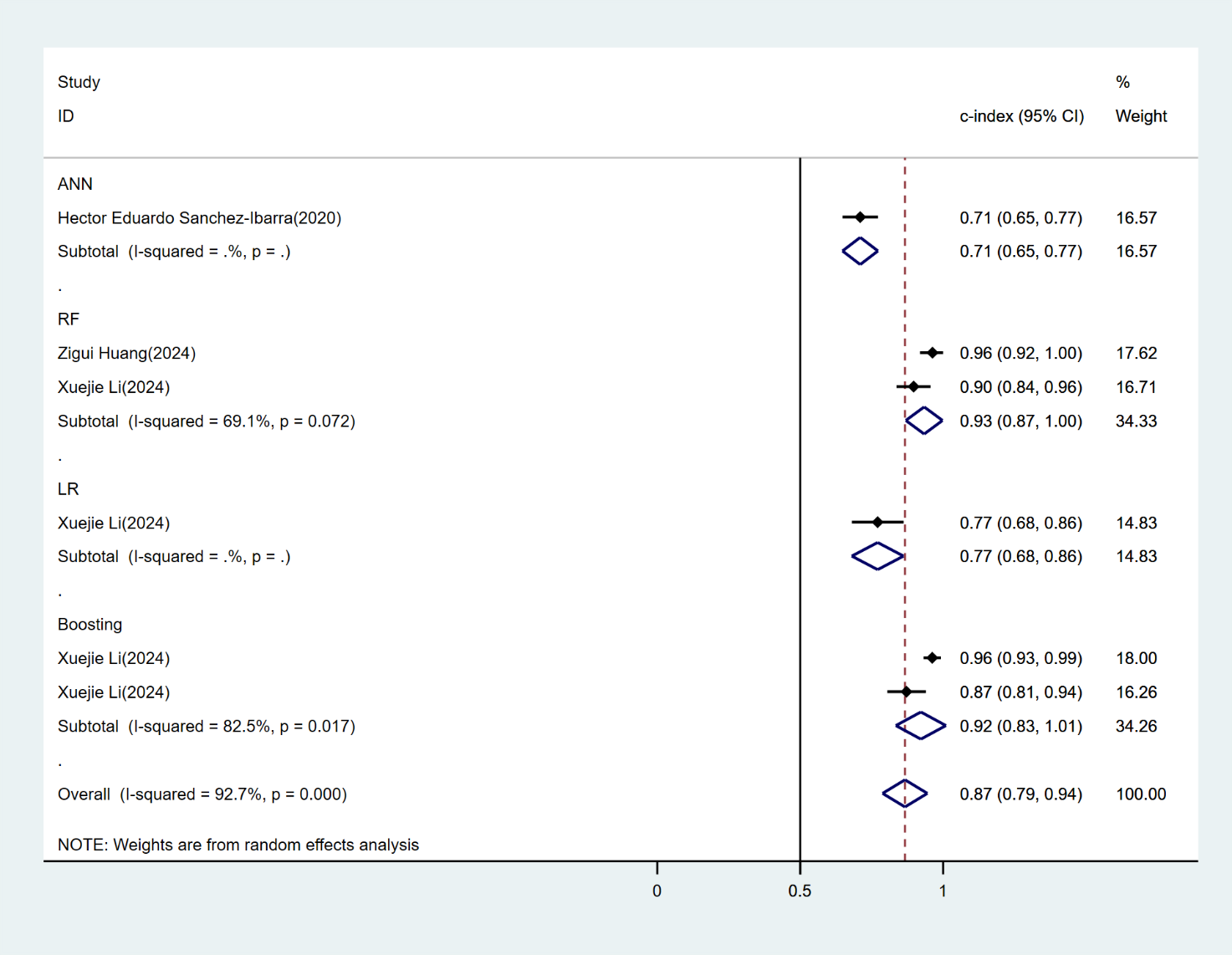


**Figure S5.** Forest plot of the c-index for detecting microsatellite instability in colorectal cancer based on pathological genomics in the training set.


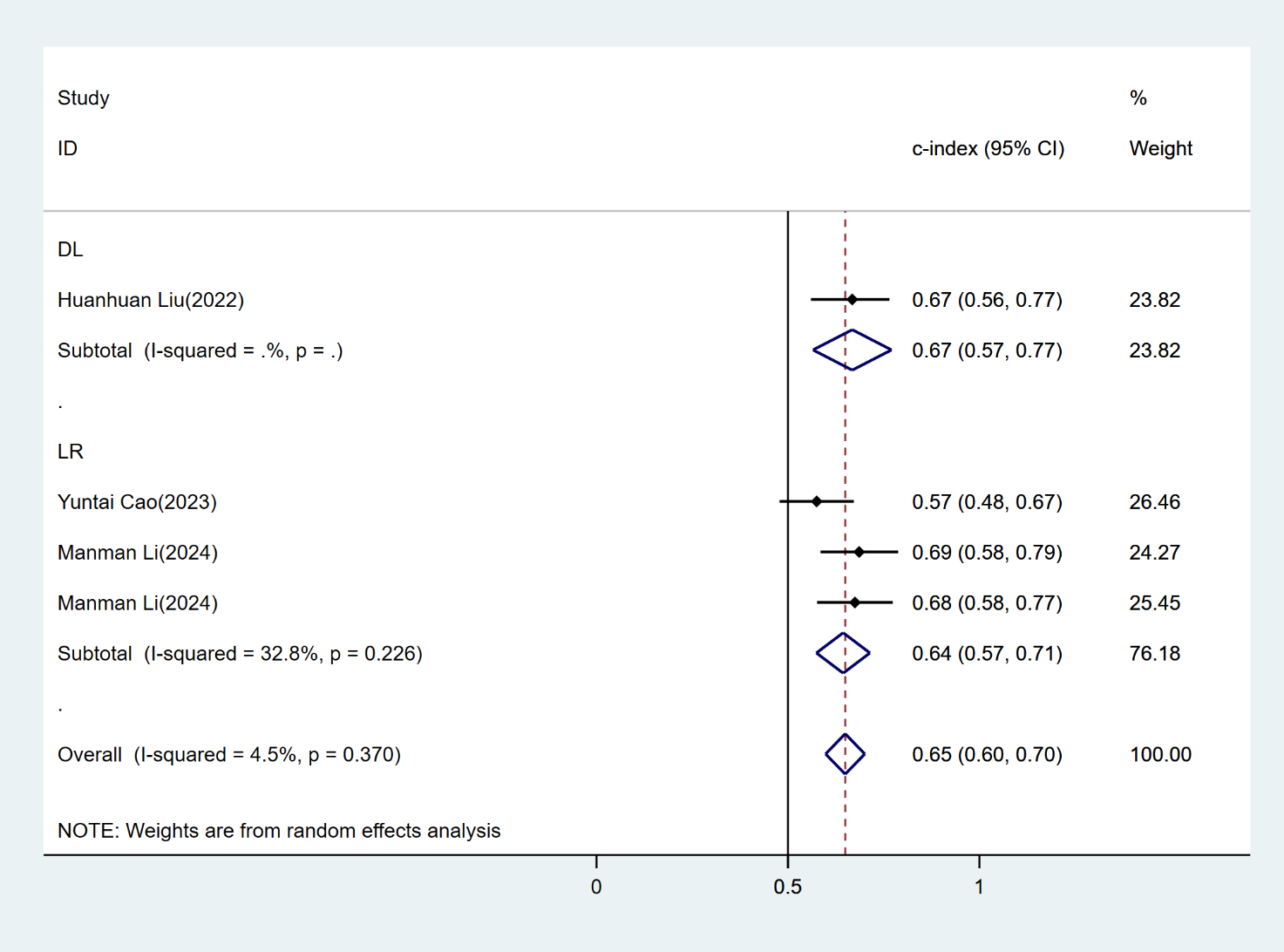


**Figure S6.** Forest plot of the c-index for detecting microsatellite instability in colorectal cancer based on clinical features in the validation set.


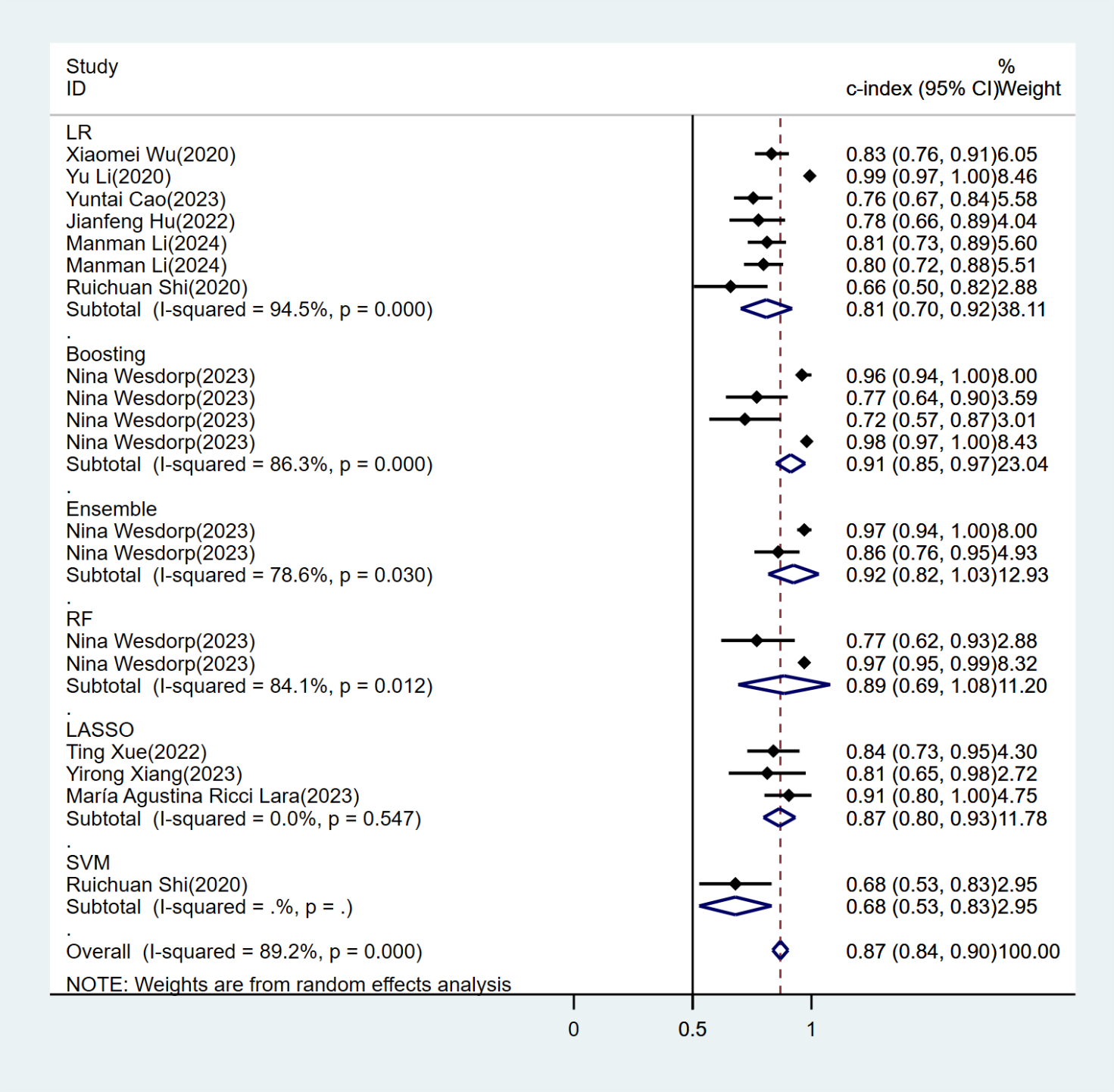


**Figure S7.** Forest plot of the c-index for detecting microsatellite instability in colorectal cancer based on CT radiomics in the validation set.


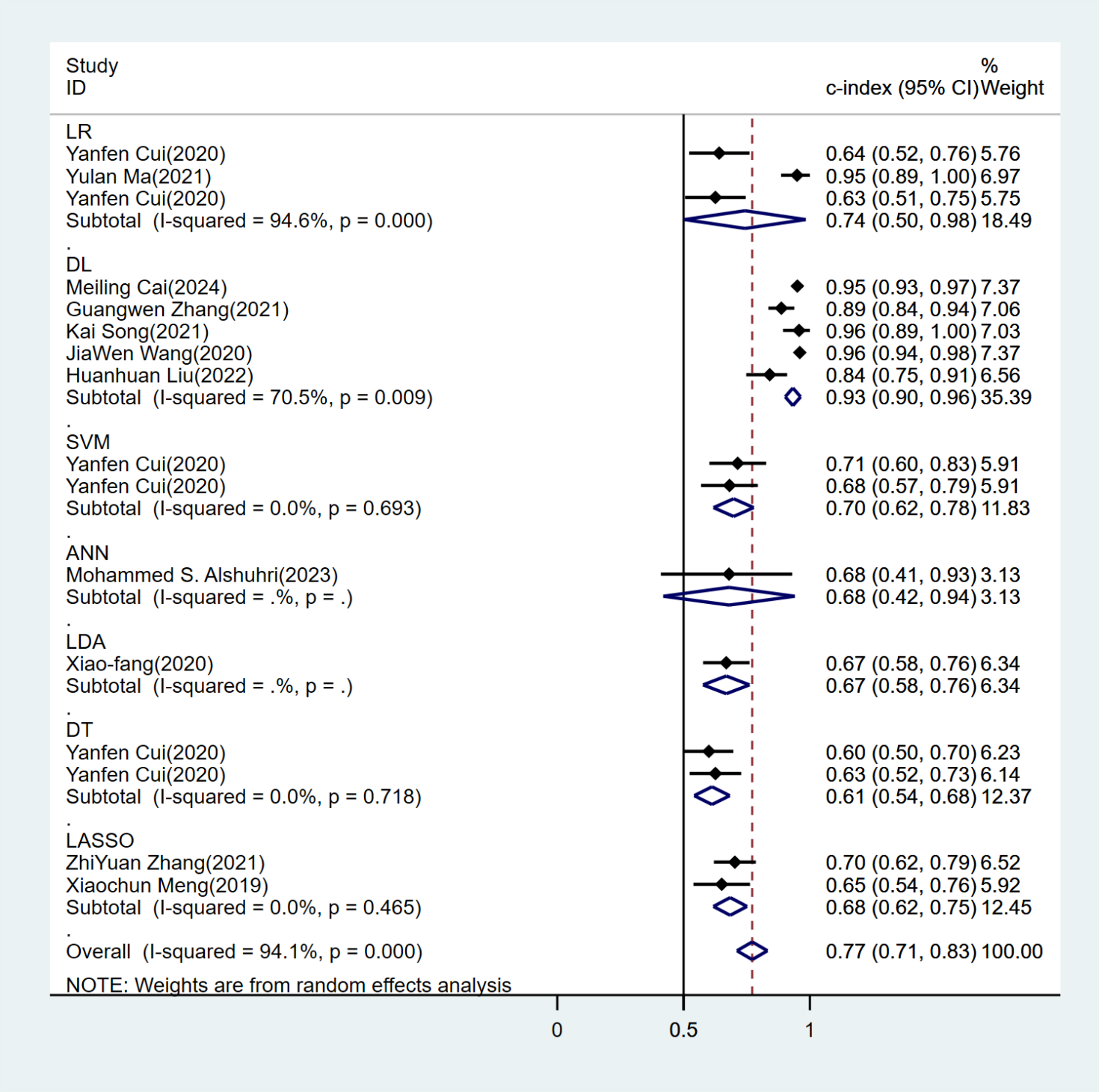


**Figure S8.** Forest plot of the c-index for detecting microsatellite instability in colorectal cancer based on MRI radiomics in the validation set.


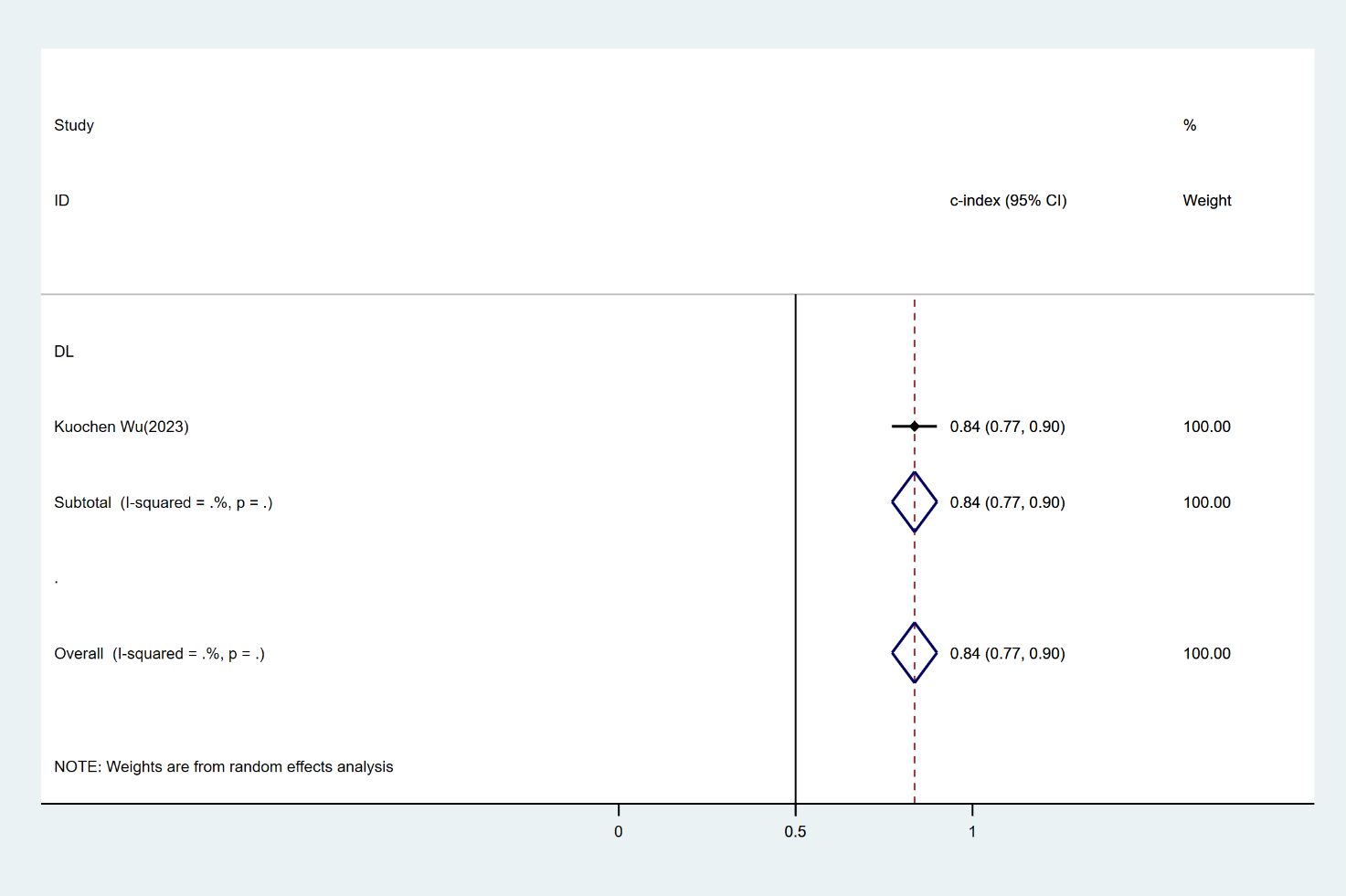


**Figure S9.** Forest plot of the c-index for detecting microsatellite instability in colorectal cancer based on PET/CT radiomics in the validation set.


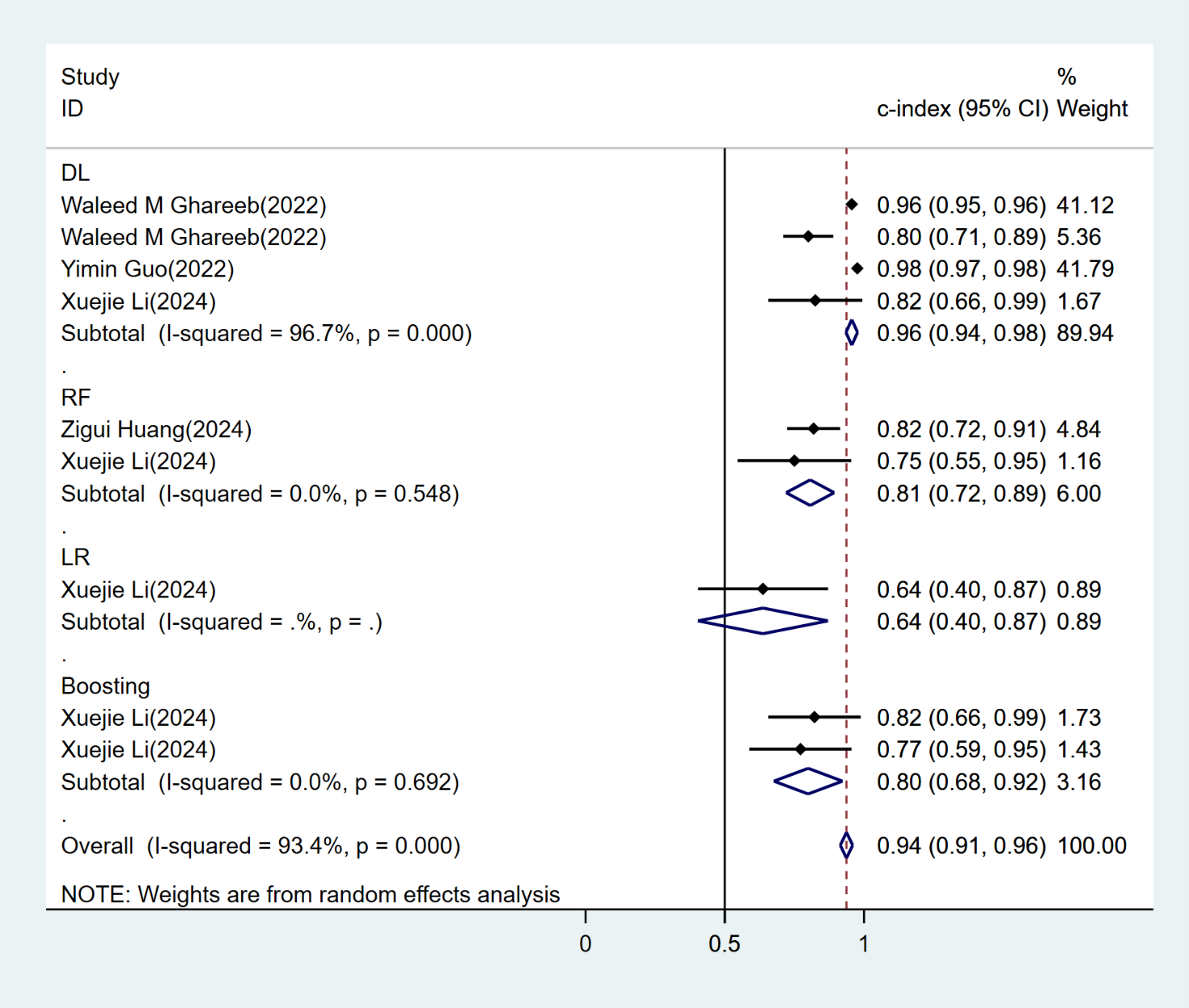


**Figure S10.** Forest plot of the c-index for detecting microsatellite instability in colorectal cancer based on pathological genomics in the validation set.


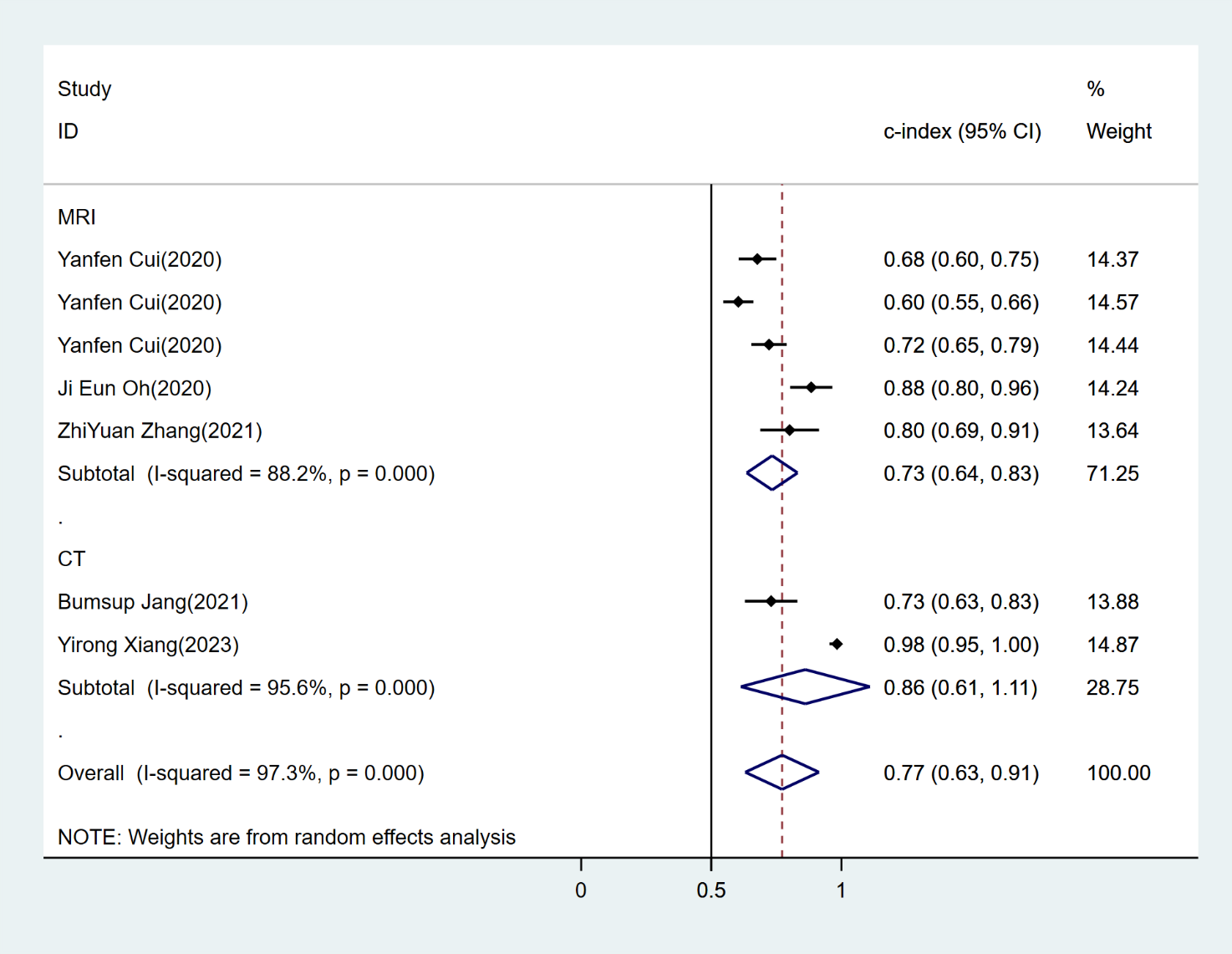


**Figure S11.** Forest plot of the c-index for microsatellite instability in rectal cancer in the training set.


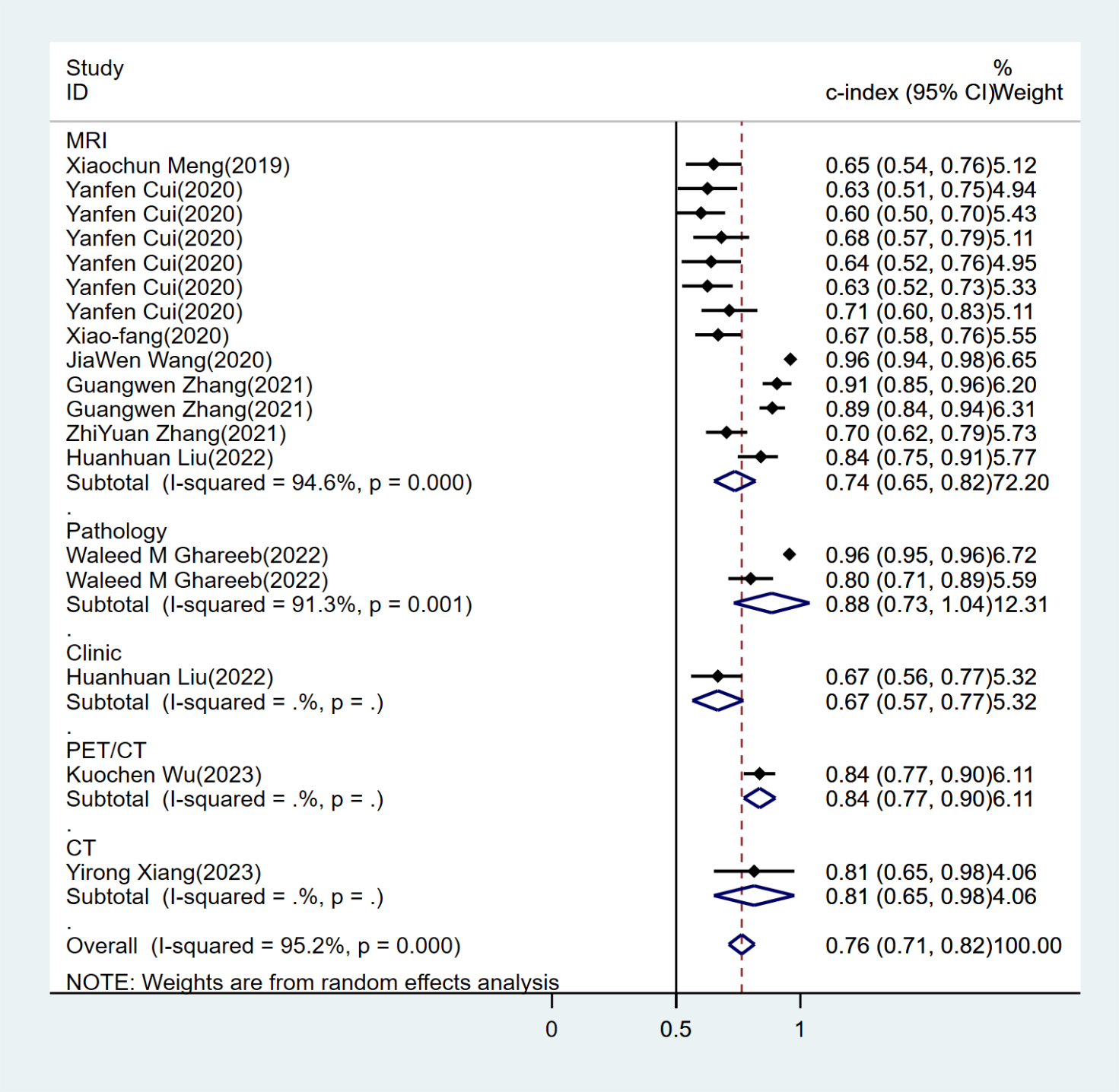


**Figure S12.** Forest plot of the c-index for microsatellite instability in rectal cancer in the validation set.


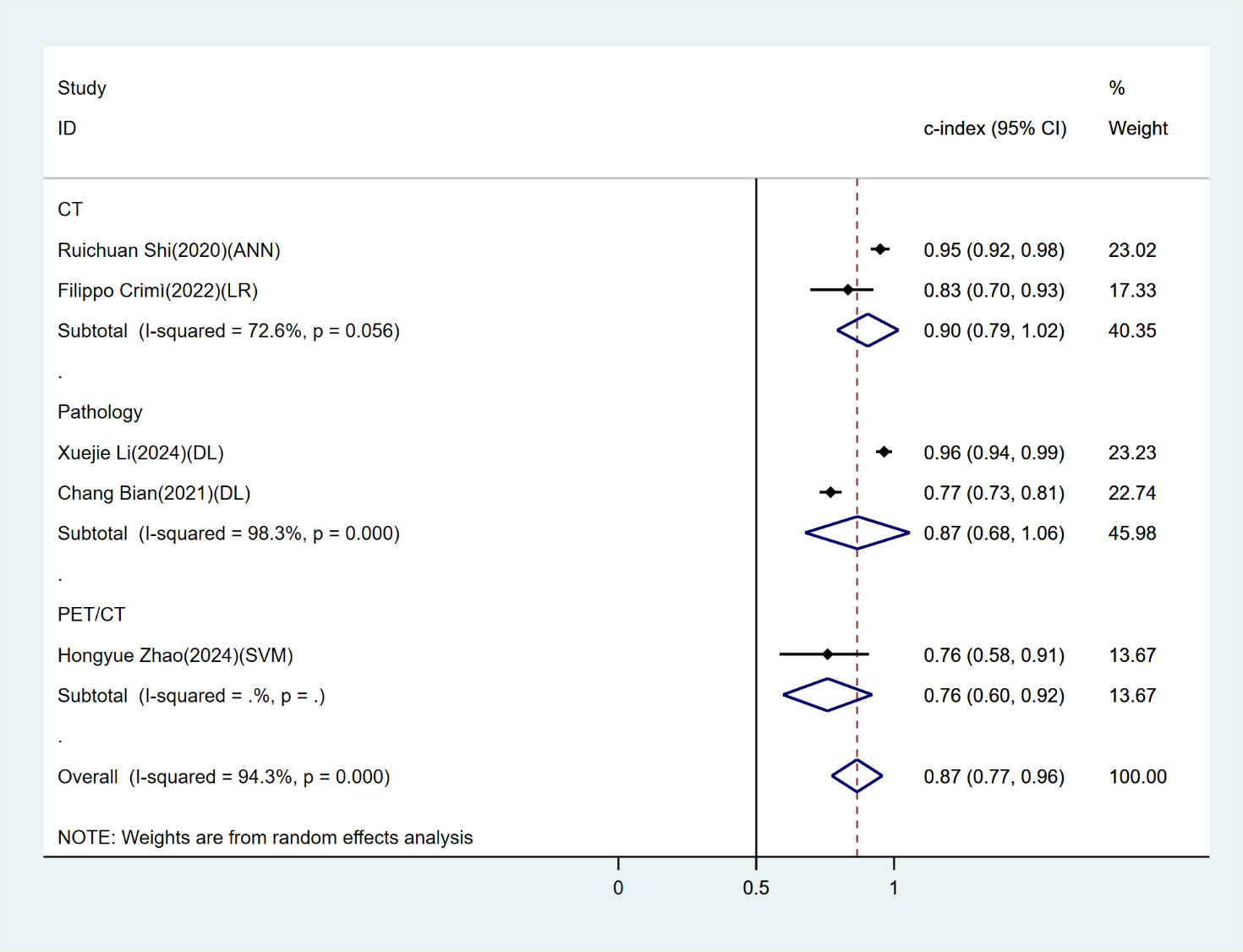


**Figure S13.** Forest plot of the c-index for mixed genotype microsatellite instability in colorectal cancer in the training set.


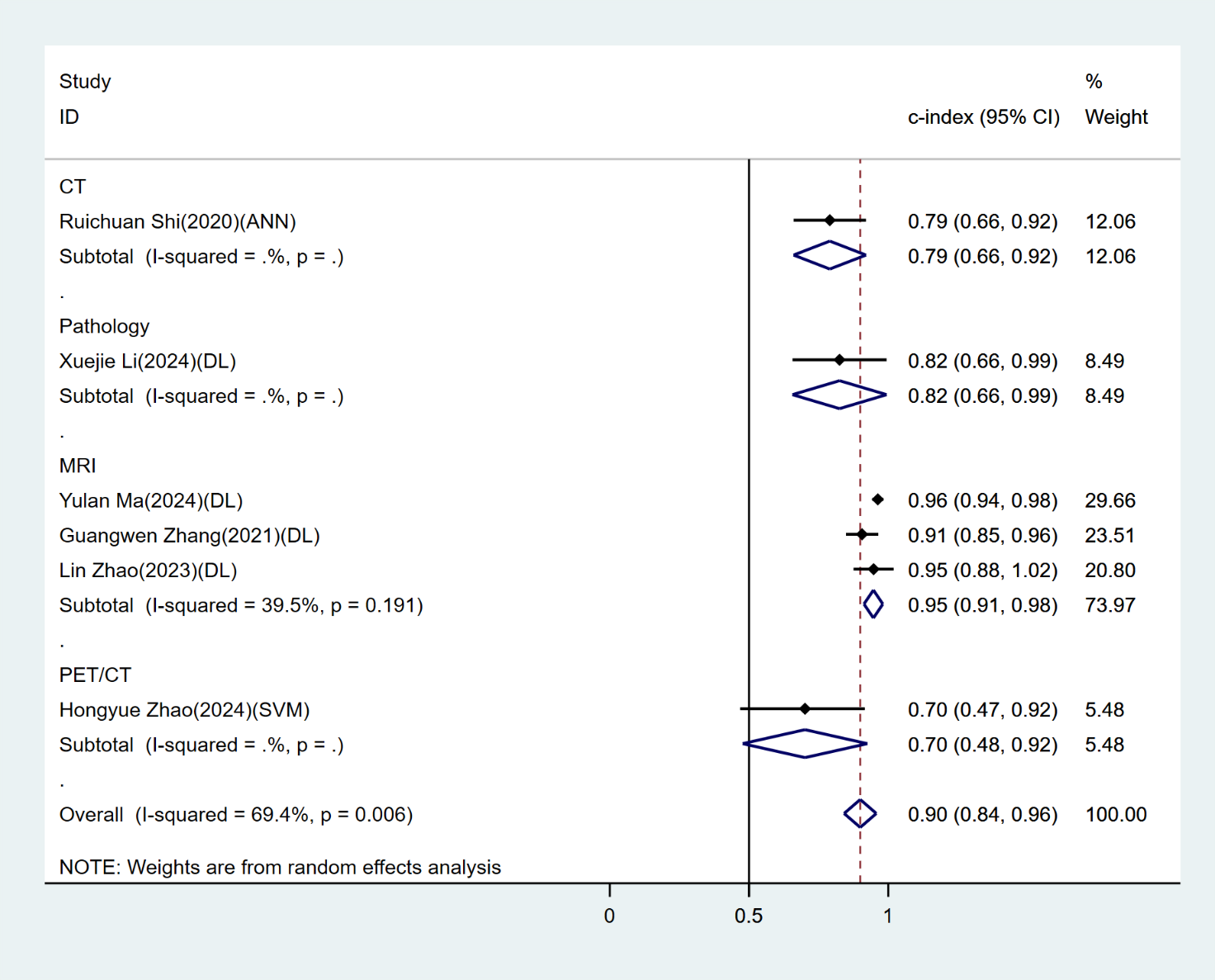


**Figure S14.** Forest plot of the c-index for mixed genotype microsatellite instability in colorectal cancer in the validation set.
